# Thermophilic Composting of Human Feces: Development of Bacterial Community Composition and Antimicrobial Resistance Gene Pool

**DOI:** 10.3389/fmicb.2022.824834

**Published:** 2022-02-18

**Authors:** Katharina A. Werner, Anja Poehlein, Dominik Schneider, Khaliel El-Said, Michael Wöhrmann, Isabel Linkert, Tobias Hübner, Nicolas Brüggemann, Katharina Prost, Rolf Daniel, Elisabeth Grohmann

**Affiliations:** ^1^Faculty of Life Sciences and Technology, Department of Microbiology, Berliner Hochschule für Technik, Berlin, Germany; ^2^Göttingen Genomics Laboratory, Department of Genomic and Applied Microbiology, Institute of Microbiology and Genetics, Georg-August-Universität Göttingen, Göttingen, Germany; ^3^Department of Environmental Microbiology, Helmholtz-Centre for Environmental Research GmbH - UFZ, Leipzig, Germany; ^4^Institute of Bio- and Geosciences Agrosphere (IBG-3), Forschungszentrum Jülich, Jülich, Germany

**Keywords:** compost, ecological sanitation, human feces, biochar, bacterial communities, bacterial isolates, antibiotic resistance genes, next-generation sequencing

## Abstract

In times of climate change, practicing sustainable, climate-resilient, and productive agriculture is of primordial importance. Compost from different resources, now treated as wastes, could be one form of sustainable fertilizer creating a resilience of agriculture to the adverse effects of climate change. However, the safety of the produced compost regarding human pathogens, pharmaceuticals, and related resistance genes must be considered. We have assessed the effect of thermophilic composting of dry toilet contents, green cuttings, and straw, with and without biochar, on fecal indicators, the bacterial community, and antibiotic resistance genes (ARGs). Mature compost samples were analyzed regarding fecal indicator organisms, revealing low levels of *Escherichia coli* that are in line with German regulations for fertilizers. However, one finding of *Salmonella* spp. exceeded the threshold value. Cultivation of bacteria from the mature compost resulted in 200 isolates with 36.5% of biosafety level 2 (BSL-2) species. The majority is known as opportunistic pathogens that likewise occur in different environments. A quarter of the isolated BSL-2 strains exhibited multiresistance to different classes of antibiotics. Molecular analysis of total DNA before and after composting revealed changes in bacterial community composition and ARGs. 16S rRNA gene amplicon sequencing showed a decline of the two most abundant phyla *Proteobacteria* (start: 36–48%, end: 27–30%) and *Firmicutes* (start: 13–33%, end: 12–16%), whereas the abundance of *Chloroflexi*, *Gemmatimonadetes*, and *Planctomycetes* rose. Groups containing many human pathogens decreased during composting, like *Pseudomonadales*, *Bacilli* with *Bacillus* spp., or *Staphylococcaceae* and *Enterococcaceae*. Gene-specific PCR showed a decline in the number of detectable ARGs from 15 before to 8 after composting. The results reveal the importance of sufficiently high temperatures lasting for a sufficiently long period during the thermophilic phase of composting for reducing *Salmonella* to levels matching the criteria for fertilizers. However, most severe human pathogens that were targeted by isolation conditions were not detected. Cultivation-independent analyses also indicated a decline in bacterial orders comprising many pathogenic bacteria, as well as a decrease in ARGs. In summary, thermophilic composting could be a promising approach for producing hygienically safe organic fertilizer from ecological sanitation.

## Introduction

Declining natural resources, high energy demand of mineral fertilizer production and wastewater treatment, as well as increasing soil degradation raise the need for alternative ideas regarding sanitation and agriculture (Mortimer et al., 2003; Yang et al., 2003; Dawson and Hilton, 2011). Ecological sanitation using thermophilic composting could tackle these problems because human excrements comprise large contents of phosphorus and nitrogen (Rose et al., 2015). Used as organic fertilizer in agriculture, compost increases humus and nutrient contents and thereby enhances soil fertility and crop yields (Hernando et al., 1989; García-Gil et al., 2004; Montemurro et al., 2006; Walter et al., 2006; Seyedbagheri, 2010; Selim and Ali Mosa, 2012; Barton et al., 2016; Solaiman et al., 2019).

Beneficial effects on soils have also been described for biochar, which is charcoal derived from organic matter through pyrolysis. In contrast to charcoal, however, biochar is produced not as a fuel but as an amendment for agriculture (e.g., for soil, compost, and animal feed) (Kimetu et al., 2008; Spokas et al., 2009; Anderson et al., 2011; Lehmann and Joseph, 2015). Addition of biochar to compost results in an acceleration of the process through better aeration, higher composting temperatures, and a longer duration of the thermophilic phase (Godlewska et al., 2017; Sanchez-Monedero et al., 2018).

However, there are major concerns regarding the safety of compost from ecological sanitation when used for crop production, concerning especially the hygiene, as well as the fate of pharmaceutical residues and antibiotic resistances (Schönning and Stenström, 2004).

Outbreaks of infectious diseases spread *via* contaminated vegetables, e.g., the EAHEC outbreak *via* sprouts between May and July 2011 with almost 4,000 infected persons in Germany only (Brzuszkiewicz et al., 2011; Robert Koch Institut [RKI], 2011), emphasize the importance of safe food products. Pathogenic strains may reach the fields *via* contaminated organic fertilizer and can survive for a long time on vegetables (Solomon et al., 2002; Islam et al., 2004; Jechalke et al., 2019). It is therefore important that fields and fertilizers are free of human pathogens. Whereas mineral fertilizers hold minimal risks, organic fertilizers such as manure or sewage sludge are potential sources of contamination and should be used with care or prior treatment. Thermophilic composting has been shown to eliminate most bacteria adapted to mesophilic temperatures of the human body, when the composting process is conducted properly (Shepherd et al., 2007).

Thermophilic composting consists of separate phases with different predominating organisms. A short initial mesophilic phase is followed by a thermophilic phase, which may last between days and weeks. Bacteria dominate the thermophilic phase, whereas fungi and yeasts are less thermotolerant and grow up again in the second mesophilic and maturation phase (lasting weeks to months). Although composting parameters are diverse and results differ, *Firmicutes* and *Proteobacteria*, along with *Actinobacteria*, *Bacteroidetes*, or *Planctomycetes* dominate all phases of composting (Ryckeboer et al., 2003; Esperón et al., 2020).

Acquired antibiotic resistance and the accumulation of multiple resistances in pathogenic bacteria are a growing health problem causing an estimated minimum of 700,000 deaths every year (Laxminarayan et al., 2013; O’Neill, 2014). There are indications that transfer of ARGs does not only take place from clinics to the environment, but also the other way around, from environmental to clinical strains (Lartigue et al., 2006; Walsh, 2006; Knapp et al., 2010; Graham et al., 2016). Although bacteria from other habitats might not survive in the soil (Heuer et al., 2008), horizontal gene transfer to soil bacteria can increase the pool of resistances in environmental habitats. Manure as a hotspot of mobile genetic elements harboring many ARGs was found to increase the resistome when applied to soils (Smalla et al., 2000; Jechalke et al., 2014; Blau et al., 2017). Moreover, some antibiotics are stable in manure and thereby possibly maintain selective pressure (Christian et al., 2003; Heuer et al., 2008; Lamshöft et al., 2010; Wolters et al., 2016). In general, the abundance of ARGs has increased in soils over the past decades, as shown in studies analyzing soil samples taken from 1923 and 1940, respectively, until now (Knapp et al., 2010; Graham et al., 2016). In comparison with soils under inorganic fertilization, manure application significantly increased the load of beta-lactam-ARGs (Knapp et al., 2010). Thermophilic composting has been suggested as one option for pre-treatment of manure and sewage, when used as agricultural fertilizer (Pruden et al., 2013; Jechalke et al., 2014; Lima et al., 2020). Besides a reduction in viable pathogenic bacteria, thermophilic composting of manure has proven to be effective in decreasing ARGs, as well. This decreasing effect was shown for beta-lactam, tetracycline, macrolide, quinolone, and sulfonamide resistance genes (Wang et al., 2016; Guo et al., 2017; Chen Z. et al., 2018; Esperón et al., 2020). However, reduction efficiencies vary strongly and some studies even show increases in single ARGs analyzed (often, but not exclusively, sulfonamide ARGs) (Li et al., 2017; Cheng et al., 2019; Duan et al., 2019; Esperón et al., 2020). Beneficial effects on the reduction of ARGs have been described for biochar and biochar-like amendment to manure compost (Li et al., 2017; Lu et al., 2018). Studies on the effect of human waste composting on ARGs, however, are scarce (Ezzariai et al., 2018). Results on the fate of ARGs during thermophilic composting of sewage sludge are not conclusive; strong variations in the applied composting processes, substrates, and treatments might be one reason (Zhang et al., 2016; Jang et al., 2018; Liao et al., 2018; Cui et al., 2020; Wei et al., 2020). Studies on the composting of dry toilet contents have thus far only focused on the reduction of pathogenic microorganisms (Tønner-Klank et al., 2007; Germer et al., 2010; Mehl et al., 2011; Sossou et al., 2014).

To assess the hygienization potential of thermophilic composting for ecological sanitation, especially regarding human pathogenic bacteria and ARGs, we have conducted composting trials with human excrements together with other organic wastes. In addition, the effect of biochar as a co-substrate during the composting process was evaluated.

## Materials and Methods

### Composting Trial and Sampling

Composting trials were conducted in Eckernförde, Northern Germany, between September 2018 and January 2019. The composition of the composting substrates is described in Supplementary Table 1. Moreover, total and volatile solids, as well as *C*/*N*-ratio of the substrates are shown in Supplementary Table 2. The biochar used in this study was produced in an open kiln reactor [“Kontiki”-type (Cornelissen et al., 2016)] from wood collected from branches and twigs from different types of trees (purchased from “Dein Stück Erde UG,” Stuttgart, Germany). Properties of the biochar used were as follows (*n* = 3): 459 ± 10 kg/m^3^ density; 37.1 ± 1.1% dry matter; 79.1 ± 10.6% volatile solids; pH 9.4 ± 0.1; 0.5 ± 0.1 mS/cm conductivity; 72.0 ± 5.6% dry matter carbon; 0.57 ± 0.07% dry matter nitrogen; 1.84 ± 0.24% dry matter hydrogen; 0.07 ± 0.01% dry matter sulfur. The high carbon content of around 70% of dry matter and high amount of volatile solids of around 80% dry matter match the properties of biochar (Lehmann and Joseph, 2009). Polycyclic aromatic hydrocarbon (PAH) analysis showed levels below 1 mg/kg dry matter for all PAHs according to EPA 16 list. Brimful 60 L buckets of biochar were added, and the dry matter of added biochar was calculated by means of the density and dry matter content of the biochar. Compost piles were turned every 2 weeks during the first 2 months of composting and once a month afterward until the end of the trial.

The trial comprised four piles and two treatments. Each of the two treatments (with and without biochar) was set up twice at an interval of 2 weeks, with E1 and E1-B referring to the replicates, which were set up first, and E2 and E2-B referring to the replicates set up 2 weeks later. “-B” indicates the biochar treatment. E1 and E1-B were set up on August 15, 2018 and E2 and E2-B set up on August 29, 2018. The dimensions of the piles as measured from the ground (length × width × height) were 6 m × 3 m × 0.9 m for E1, 5 m × 3 m × 0.9 m for E1-B, 8 m × 3 m × 0.8 m for E2, and 5 m × 2 m × 0.8 m for E2-B. Moisture of the compost material on the day of experimental set-up was 61, 64.3, 66.5, and 67.1% for E1, E1-B, E2, and E2-B, respectively. Temperature curves of the averaged measurements are shown in Supplementary Figure 1, and data are listed in the Supplementary Table 3.

Sampling was conducted at the day of the experimental set-up (“start” samples), after 2 weeks (only repetition 1), and at the end of the experimental trials after 5.5 (repetition 1) and 5 months (repetition 2), respectively. Samples were taken from the center of each compost pile at a depth of approximately 0.6 m at three different positions (front, middle, and back of each pile), respectively. Therefore, a hole was dug into the pile and approximately 20 L of substrate/compost were excavated and homogeneously mixed on a tarp. From this homogenous mixture circa 300 g was sampled for analysis. All samples were stored at 4°C for the isolation studies and at −20°C for molecular analyses.

### Most Probable Number Method for the Detection of *Salmonella* spp. and *Escherichia coli*

For the assessment of compost hygiene, *Salmonella* and *Escherichia coli* were detected by means of the most probable number technique (MPN) according to the German Federal Ministry for the Environment, Nature Conservation, and Nuclear Safety (Pietsch et al., 2014). Statistical estimates of MPNs of colony forming units (CFU) in the original samples were taken from the tables of De Man (1983). Triplicate samples of the center of the mature compost were analyzed directly after sampling. To detach the bacteria, 10 g of compost was suspended in 90 mL 0.9% sodium chloride solution (10^–1^ dilution) and shaken at 150 rpm at 4°C overnight. A 10-fold dilution series up to 10^–9^ was prepared. Triplicates of each dilution were applied to a series of cultivation steps as follows.

For *Salmonella*, 1 mL of each dilution was added to a test tube with 9 mL buffered peptone water (Carl Roth GmbH), vortexed for 10 s, and incubated at 37°C for 24 h ± 1 h. A total of 100 μL of this enrichment was transferred into 10 mL Rappaport-Vassiliadis broth (Carl Roth GmbH, Karlsruhe, Germany), vortexed for 10 s, and incubated at 42°C for 24 h ± 1 h. One loopful of the sample was spread on two specific agars for identification: brilliant green-lactose-sucrose agar (BPLS, Carl Roth GmbH, Karlsruhe, Germany) and xylose-lysine-deoxycholate agar (XLD, Carl Roth GmbH, Karlsruhe, Germany). Plates were incubated at 37°C for 24 h ± 1 h. Putative positive colonies were transferred to standard-I-agar [15 g/L peptone ex casein, 6 g/L NaCl, 3 g/L yeast extract, 1 g/L D (+) Glucose, 12 g/L agar-agar], and incubated at 37°C overnight for verification *via* slide agglutination test with the monoclonal test serum “Anti-Salmonella A-67 + Vi, omnivalent” (Sifin Diagnostics GmbH, Berlin, Germany) according to the manufacturer’s instructions.

Detection of *E. coli* was performed in parallel from the same dilution series. One milliliter of each dilution transferred to 9 mL MacConkey broth (Carl Roth GmbH, Karlsruhe, Germany), vortexed for 10 s, and incubated at 37°C for 24 h ± 1 h. If a color change to yellow, indicating lactose fermentation, was observed, samples were streaked on MacConkey agar (Carl Roth GmbH, Karlsruhe, Germany) for verification.

Both *Salmonella* and *E. coli* results were documented indicating the confirmed positive (+) or negative (−) results for each of the triplicates in each dilution step. From the number of positive replicates in the highest three dilutions showing positive results, an index number was generated to be used with the respective tables of De Man (1983).

### Isolation and Identification of Bacterial Isolates

From the mature compost, bacterial isolates were obtained. Samples from the front and the back of each pile were used for isolation, resulting in eight samples in total. Samples were diluted 1:10 (w/v) in 0.9% sodium chloride solution and shaken overnight at 4°C and 150 rpm. Afterward, a 10-fold dilution series was prepared. One hundred μL of each dilution (10^–2^ – 10^–7^) were plated in triplicates on R2A agar and CHROMagar™ ESBL (Mast Diagnostica GmbH, Reinfeld, Germany), respectively. Plates were incubated at 37°C overnight. Colonies were classified according to their morphology and subsets of each group transferred to selective agar for specific isolation of severe human pathogenic bacteria (Supplementary Table 4).

Isolates were identified *via* matrix-assisted laser desorption ionization time-of-flight mass spectrometry (MALDI-TOF MS, Bruker Daltonics MALDI Biotyper system) using the protein extraction procedure according to the manufacturer’s instructions (Bruker MALDI Biotyper 3.1 User Manual Revision 4, 2015). Mass spectra were compared with the MALDI-BDAL Database (Version 3.1). Scores between 2.0 and 3.0 were interpreted as high-confidence identification as recommended by the manufacturer. Lower scores were considered not sufficient for identification.

All by mass spectrometry not sufficiently identified and all BSL-2 isolates with a MALDI score below 2.3 were subjected to 16S rRNA gene Sanger sequencing (Eurofins Genomics Germany GmbH, Ebersberg, Germany). For this, single colonies of the isolates were suspended in 300 μL sterile distilled water and incubated at 95°C for 15 min to disrupt the cells. There was 0.5 μL template used in a 10 μL PCR reaction containing 0.2 U Taq polymerase (peqGOLD, VWR), 1 x PCR reaction buffer S (peqGOLD, VWR), 0.2 μM of each primer (16S rRNA gene standard primers 27f and 1492r), 250 μM of each dNTP, and 1 μg bovine serum albumin (BSA). PCR was initiated by heating to 95°C for 5 min, followed by 30 cycles of 30 s at 95°C, 30 s at 50°C, and 10 s at 72°C. The final elongation step was carried out for 7 min at 72°C. The PCR products were sequenced by Eurofins Genomics Germany GmbH, Ebersberg, Germany. Sequences were aligned against the nucleotide database (nt) of BLAST sequence analysis tool (Altschul et al., 1990).

### Antibiotic Disk Diffusion Assay

Disk diffusion assays (disks from Oxoid™) were performed to analyze the antibiotic resistance of the isolates. Sixteen frequently used antibiotics, ampicillin, meropenem, imipenem, ciprofloxacin, erythromycin, vancomycin, clindamycin, doxycycline, tetracycline, tigecycline, kanamycin, gentamicin, sulfamethoxazole, norfloxacin, ofloxacin, and nitrofurantoin were selected for analysis according to the guidelines of the European Committee on Antimicrobial Susceptibility Testing (EUCAST^1^). Isolates were tested against the antibiotics recommended by EUCAST for the respective genus (Supplementary Table 5). Respective breakpoints were used for interpretation of results (EUCAST, 2019). Since EUCAST does not provide breakpoints for *Achromobacter* spp., *Bacillus* spp., and *Brucella* spp., breakpoints were derived from relevant publications (Supplementary Table 5). Each test was performed in triplicate on Mueller Hinton agar. Results were documented after 16–20 h of incubation at 37°C.

*Brucella intermedia* was tested according to the method described by Gu et al. (2020). Pretesting of isolates identified an OD_600_ of 0.1 in phosphate-buffered saline (0.137 mM NaCl, 2.7 mM, KCl, 10 mM, Na_2_HPO_4_, 1.8 mM KH_2_PO_4_, pH 7.2) as equivalent to a cell number of 1 × 10^8^ CFU/mL, which was applied in the assay accordingly. CASO agar with 10% newborn calf serum as suggested by the authors was tested in comparison with CASO agar without the serum. Since CFUs did not vary for the isolates tested, CASO agar without additives was used for the assay. The plates were incubated at 37°C for 16–20 h before measuring the halo diameters.

### Sample Preparation, Total DNA Extraction, and Purification

Compost was stored at −20°C after sampling. Due to the very inhomogeneous structure, especially of the material prior to composting, pretreatment was necessary before DNA extraction. Therefore, samples were dried at 40°C until weight loss was no longer detectable (between 14 and 22 days) and grinded using a vibratory disk mill (Fritsch) with an agate p-9 grinding set for 5 min at 750 rpm. Grinding jars were cleaned after each sample through a 2-min grinding step with quartz sand and subsequent washing.

Total DNA was extracted from 250 mg compost samples using the NucleoSpin^®^ Soil gDNA extraction kit (Macherey-Nagel) according to the manufacturer’s protocol. Lysis buffer SL2 was applied without enhancer. For the mechanical disruption of the samples a FastPrep-24™ 5G homogenizer (MP Biomedicals) was used at 5 m/s for 30 s. Extracts were eluted in 50 μL nuclease-free water, purified using a magnetic separation device NucleoMag SEP (Macherey-Nagel) and CleanNGS kit (CleanNA) according to the manufacturer’s instructions and stored at −20°C.

### Analysis of Antimicrobial Resistance Genes

Thirty-two different ARGs were searched for using PCR with gene-specific primers (Supplementary Table 6). Every PCR reaction contained 0.025 U/μL Taq polymerase (peqGOLD, VWR), 1 × PCR reaction buffer S (peqGOLD, VWR), 0.2 μM of each primer, 200 μM of each dNTP, 1–5 ng template. All PCR programs started with an initial denaturation (95°C, 2 min) and end with a final elongation (72°C, 5 min) step (Supplementary Table 7). Results were evaluated by agarose gel electrophoresis on 1.5% agarose gels and documented as “−” (no band visible) and “+” (band visible) for each sample.

### Illumina Sequencing

#### 16S rRNA Gene Amplicon PCR Assays

For Illumina sequencing, 16S rRNA gene amplicons were generated in a two-step PCR approach using primers 341f (5′-CCTACGGGNGGCWGCAG-3′) and 805r (5′- GACTACHVGGGTATCTAATCC -3′) without adapters in a first round to generate considerable amounts of PCR product. The PCR product was used as a template in a second round with primers S-D-Bact-0341-b-S-17 (5′-TCGTCGGCAGCGTCAGATGTGTATAAGAGACAGCCTACG GGNGGCWGCAG-3′) and S-D-Bact-0785-a-A-21 (5′- GTCTCGTGGGCTCGGAGATGTGTATAAGAGACAGGACTA CHVGGGTATCTAATCC -3′) (Herlemann et al., 2011; Klindworth et al., 2013). PCR was performed in triplicate per sample. For both PCR steps, the Phusion^®^ High-Fidelity PCR Kit (New England BioLabs^®^ Inc., Ipswich, MA, United States) was used with 0.02 U/μL polymerase, 1× buffer GC, 0.2 μM of each primer, 200 μM of each dNTP, 0.2 mM MgCl_2_, 5% DMSO, and 0.6 μg BSA. For the first round, 0.5 μL of the 10-fold diluted purified DNA extracts (1–5 ng) were used in 10 μL PCR reactions consisting of an initial denaturation of 5 min at 95°C, 20 cycles of 30 s at 95°C, 30 s at 50°C, and 10 s at 72°C and a final elongation of 7 min at 72°C. For the second round, 1 μL of PCR product from the first round was used as a template in a 50-μL reaction. Thermal cycling was carried out at 98°C for 1 min, followed by 8 cycles consisting of 45 s at 98°C, 45 s at 60°C, and 10 s at 72°C, and a final elongation at 72°C for 5 min. PCR products were quantified by means of agarose gel electrophoresis and purified using the NucleoMag^®^ 96 clean-up kit (Macherey-Nagel) according to manufacturer’s instructions and eluted in 50 μL of sterile, nuclease-free water. Triplicates were pooled in equal volumes for sequencing.

#### Library Preparation and Sequencing

Indices and Illumina sequencing adapters were attached to the PCR products using the Nextera XT Index kit (Illumina, San Diego, CA, United States). Index PCR was performed using 5 μl of template PCR product, 2.5 μl of each index primer, 12.5 μl of 2× KAPA HiFi HotStart ReadyMix, and 2.5 μl PCR grade water. The thermal cycling scheme was as follows: 95°C for 3 min, 8 cycles of 30 s at 95°C, 30 s at 55°C and 30 s at 72°C and a final extension at 72°C for 5 min. Quantification of the products was performed using the Quant-iT dsDNA HS assay kit and a Qubit fluorometer (Invitrogen GmbH, Karlsruhe, Germany) following the manufacturer’s instructions. MagSi-NGS*^PREP^* Plus Magnetic Beads (Steinbrenner Laborsysteme GmbH, Wiesenbach, Germany) were used for purification of the indexed products as recommended by the manufacturer. Normalization of all libraries to the same concentration was performed using the Janus Automated Workstation from Perkin Elmer (PerkinElmer, Waltham, MA, United States). Sequencing was conducted using Illumina MiSeq platform using dual indexing and MiSeq reagent kit v3 (600 cycles) as recommended by the manufacturer.

#### Sequence Processing and Analyses

Demultiplexing and clipping of adapter sequences from the raw amplicon sequences were performed with the CASAVA software (Illumina). The program fastp (v0.20.0) (Chen S. et al., 2018) with a minimum phred score of 20, a minimum length of 50 base pairs, a sliding window size of four bases, read correction by overlap, and adapter removal of the Illumina Nextera primers was used. Paired-end reverse reads were merged with the paired end read merger (PEAR v.0.9.11) (Zhang et al., 2014) with default settings. Additionally, reverse and forward primer sequences were removed with cutadapt (v2.5) (Martin, 2011) with default settings. Sequences were then size-filtered (≤300 bp were removed) and dereplicated by vsearch (version 2.14.1; Rognes et al., 2016). Denoising was performed with the UNOISE3 module of vsearch and a set minimum size of eight reads. Chimeric sequences were excluded with the UCHIME module of vsearch. This included *de novo* chimera and reference-based chimera removal against the SILVA SSU 138 NR database (Quast et al., 2013; Bolyen et al., 2019). Sequences were mapped to amplicon sequence variants (ASVs) by vsearch with a set identity of 0.97. Taxonomy assignments were performed with BLASTn (Altschul et al., 1990, version 2.9.0+) against the SILVA SSU 138 NR database with an identity threshold of 90%. We used identity and query coverage to mark uncertain blast hits. As recommended by the SILVA ribosomal RNA database project, we removed the taxonomic assignment for blast hits with (pident + qcovs)/2 ≤ 93. Abundance bar charts were created from ASVs with the ggplot2 package with R (R Core Team, 2021) and RStudio^®^ (Wickham, 2016). Heatmap and non-metric multidimensional scaling (NMDS) plots were prepared using the ampvis2 package in RStudio^®^ (Andersen et al., 2018).

#### Nucleotide Sequence Accession Numbers

16S rRNA gene amplicon sequences were submitted to the NCBI Sequence Read Archive4 (SRA) under the NCBI BioProject study number SRP347939 with BioProject accession number PRJNA782422. A detailed list of all accession codes is given in the Supplementary Table 8.

## Results

### Hygienic Assessment of Mature Compost

Triplicates of mature compost were analyzed by culture methods for the presence of *Salmonella* spp. and *E. coli* as indicator organisms for compost hygiene. *Salmonella* was detected in one triplicate of pile E1, resulting in a mean of <120 CFU/g for E1. *E. coli* was present in all piles in small numbers (Supplementary Table 9).

### *Bacillus* and *Brucella* spp. Dominate Within the Targeted Bacterial Groups in Mature Compost

Bacteria were isolated from the mature compost (two replicates per pile) through targeted cultivation on bacterial groups containing human pathogens. In total, 200 isolates were obtained and phylogenetically affiliated *via* MALDI-TOF MS biotyping and Sanger sequencing of the 16S rRNA gene. Within these groups, the isolates were assigned to 14 genera, *Bacillus* being the largest group with 37% of isolates. 24.5% of the isolates belong to *Brucella* spp., 12% to *Serratia* spp., and 10% to *Pseudomonas* spp. Further genera comprise *Bordetella* (7%), *Achromobacter* (2.5%), *Stenotrophomonas* (2.5%), *Staphylococcus* (1%), and *Streptomyces* (1%). In addition, *Castellaniella, Cellulosimicrobium, Dermacoccus, Lysinibacillus*, and *Rhodococcus* are represented by one isolate each. The number of isolates per compost pile varied between 33 and 63, but numbers derived from biochar and non-biochar treatments were similar (biochar: 96, non-biochar: 104). No clear patterns were observed regarding distribution of genera and abundances in biochar-containing piles and biochar-free ones. *Serratia* was an exception, with 18 isolates from the biochar-containing piles and 6 from the non-biochar ones. Although less abundant, *Bordetella* (biochar: *n* = 5, non-biochar: *n* = 9) and *Stenotrophomonas* (biochar: *n* = 1, non-biochar: *n* = 4) also showed notable differences in their distribution between biochar and non-biochar treatments. In the non-biochar treatments, a higher diversity of genera was found (13 vs. 9). Besides, most genera were slightly more abundant in the non-biochar treatments, except *Serratia*, *Achromobacter*, and *Lysinibacillus* spp. that were more abundant in the biochar treatments. Eleven genera were found in the first repetition and nine in the second one. Nineteen *Serratia* isolates were derived from the first composting repetition vs. five from the second repetition. *Bacillus* (55 vs. 19) also mostly occurred in the first repetition. *Pseudomonas* isolates peaked in the second repetition (14 vs. 6), as well as *Achromobacter* (5 vs. 0) and *Stenotrophomonas* (4 vs. 1). Forty different species were identified, 10 of which are assigned to BSL-2 and 3 that are not assigned to a biosafety level yet (“*Pseudomonas sediminis*,” “*Pseudomonas sihuiensis*,” “*Serratia surfactantfaciens*”). Seventy-three isolates in total belong to BSL-2 (36.5% of identified isolates), slightly more BSL-2 isolates were found in the non-biochar treatments (*n* = 39). The largest group within BSL-2 isolates consists of *B. intermedia* (formerly known as *Ochrobactrum intermedium*, *n* = 42), followed by *Serratia marcescens* (*n* = 11) and *Pseudomonas mendocina* (*n* = 6). Supplementary Table 10 gives the identified isolates and their numbers in the different samples.

Of the species of interest for which the culture conditions were selected (Supplementary Table 4: *Acinetobacter baumannii*, *Bacillus anthracis*, *Bacillus cereus*, *Enterococcus faecalis*, *Enterococcus faecium*, *Pseudomonas aeruginosa*, and *Staphylococcus aureus*) only *B. cereus* and *P. aeruginosa* could be isolated. In terms of the genera of interest, *Bacillus*, *Pseudomonas*, and *Staphylococcus* spp. were identified. Potential serious human pathogens such as *Bacillus anthracis*, *Staphylococcus aureus*, *Acinetobacter* spp., and *Enterococcus* spp. were not isolated.

### Multiple Resistances in 20.5% of Tested Bacterial Isolates

All 73 BSL-2 isolates and 12 isolates, for which the BSL is not available, were tested for antibiotic resistances by means of agar diffusion assays. Most isolates showed resistance toward at least one antibiotic, except most *Pseudomonas* isolates that were sensitive to all antibiotics tested. Fifteen isolates showed multiple resistances: one isolate of *B. cereus*, *B. intermedia*, and *Staphylococcus haemolyticus*, as well as two “*S. surfactantfaciens*,” three *S. maltophilia*, and seven *S. marcescens* isolates. Five of the multiresistant strains were isolated from the compost piles without biochar, 10 from the biochar treatments. Antibiotic resistance profiles of all tested isolates are shown in Supplementary Table 11. Supplementary Table 12 shows the BLASTn percent identity values and query coverage of isolates of the same origin and phenotypic resistance profile. Different BLAST scores could indicate different strains.

### *Proteobacteria* and *Firmicutes* Relative Abundance Declines During Composting

The total bacterial community composition was assessed by Illumina sequencing of the 16S rRNA gene amplicons from the total community DNA. DNA was extracted from the starting samples (mixed sample from each pile), 14 days samples [mixed sample from each pile; only repetition 1 (first of the two composting repetitions, see Section “Composting Trial and Sampling”)] and end samples (triplicates from each pile). We aimed to compare the bacterial community before and after composting, as well as the treatments with and without biochar as a co-substrate of the composting process.

**FIGURE 1:**
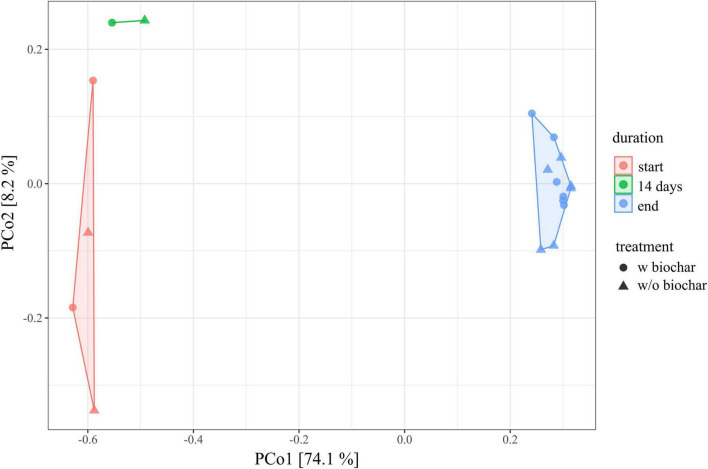
Principal coordinates analysis (PCoA) of the 16S rRNA gene amplicon data depicting the dissimilarity of each individual sample toward all others, with PCo1 (*x*-axis) explaining 74.1% differences and PCo2 (*y*-axis) explaining 8.2%, respectively. Each data point represents 1 of the 18 compost samples of the trial [E1, E1-B, E2, and E2-B from start (red), 14 days (green; only E1 and E1-B), and end (blue; triplicates per pile)]. Dots represent the treatments with biochar, triangles the ones without.

The composition of the bacterial community changed from the start to the end as depicted by principal coordinates analysis of all samples analyzed (Figure 1). Start, 14-day, and end samples form distinct clusters over the *x*-axis, which explains 74.1% of the differences observed. Fourteen-day samples are closer to the start samples, indicating higher similarity with the bacterial community from the beginning of composting than with the mature compost community.

**FIGURE 2:**
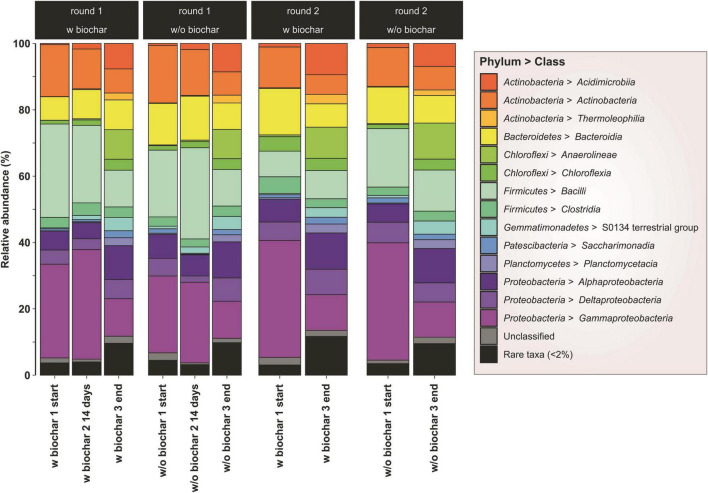
Bacterial community composition as assessed by 16S rRNA gene amplicon sequencing. Phyla are represented as percentage of the total number of sequences per sample. Samples from the start and 14 days composting derive from one mixed sample, respectively. Samples from the mature compost (end) are mean values of biological triplicates.

Most abundant phyla in both start and end samples were *Proteobacteria* [minimum and maximum values within the four compost piles; start (mixed sample per pile): 36–48% → end (mean of triplicates per pile): 27–30% of all sequences], *Firmicutes* (13–33% → 12–16%), *Actinobacteria* (13–18% → 16–18%), *Chloroflexi* (1–5% → 13–15%), *Bacteroidetes* (7–14% → 8–10%), *Gemmatimonadetes* (0.5–1.1% → 3.7–5.1%), and *Planctomycetes* (0.1–0.7% → 3.7–4.7%) (Figure 2). This corresponded with the isolation study, where all identified species were affiliated to the three most abundant groups of the sequencing results. The number of *Proteobacteria* and *Firmicutes* declined toward the end of composting, whereas *Chloroflexi*, *Gemmatimonadetes*, and *Planctomycetes* rose in numbers.

**FIGURE 3:**
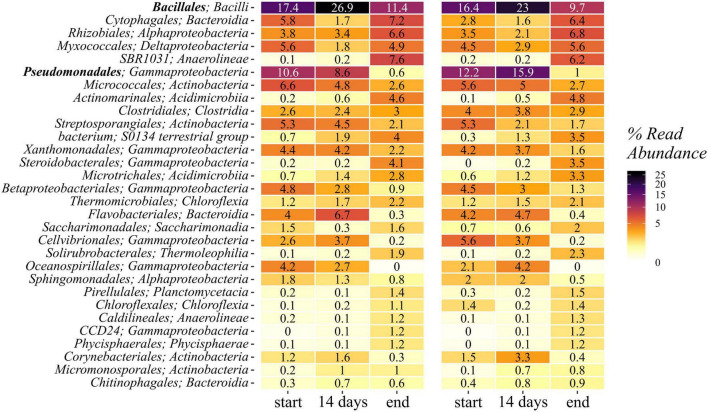
Heatmap showing the 30 most abundant classes within the 16S rRNA gene amplicon reads. Means of start, 14 days, and end samples are given for the treatments with and without biochar.

*Gammaproteobacteria* represented the most abundant group within the phylum *Proteobacteria*, followed by *Alpha*- and *Deltaproteobacteria*. The first accounted for 23–35% of the sequences in the samples from the start and declined by half toward the end of composting (11–12%). This trend was due to strong reductions in the most abundant orders *Pseudomonadales* (5–16% → 1%) and *Xanthomonadales* (3–5% → 1–2%), whereas other orders within the *Gammaproteobacteria* increased in relative abundance toward the end of composting. Among them, *Steroidobacterales* (0–0.3% → 3.1–4.2%) was the most prominent one with slightly higher numbers in the non-biochar treatments (Figure 3). The orders *Pseudomonadales*, *Xanthomonadales*, and *Steroidobacterales* contained only two different families each: *Pseudomonadaceae* and *Moraxellaceae* within the *Pseudomonadales* showed highest relative abundances in the 14-day samples and an overall decline over composting. This was due to changes in relative abundance in the genera *Pseudomonas* and *Acinetobacter*, respectively. *Xanthomonadales* were represented by *Xanthomonadaceae* with *Luteimonas* (1.7–2.9% → 0.3–0.6%) as the most abundant genus, and *Rhodobacteraceae* with *Pseudofulvimonas* as the most abundant genus (0.11–0.56% → 0–0.02%).

Despite the decline in abundance of *Pseudomonas* to 0.6–1.1% in the end samples, *Pseudomonas* spp. made up 10% of all isolates. *Serratia*, representing 12% of the isolates, were not found in the 16S rRNA gene amplicons. However, the family they belong to, the *Enterobacteriaceae*, were present (0–1.5% of total bacterial sequences).

*Alphaproteobacteria* increased in relative abundance from 6 to 7% in the start samples to 10–11% after composting. This trend was mainly visible in the most abundant order *Rhizobiales* (2.7–4.8% → 6.5–7.2%). However, the relative abundance of the most prominent familiy in both start and end samples, *Rhizobiaceae*, declined (2.3–4.1% → 1.9–2.1%), whereas the following families showed rising numbers (*Hyphomicrobiaceae*, *Devosiaceae*, *Xanthobacteraceae*).

Next in abundance were the orders *Sphingomonadales* with only one assigned family (*Sphingomonadaceae*) and a declining trend (1.2–2.4% → 0.5–0.9%) and *Rhodovibrionales*, which showed a small increase in most compost piles (0.3–0.7% → 0.5–0.6%). Interestingly, abundance of members of one of the two families declined (*Fodinicurvataceae*), whereas numbers of the other rose (*Kiloniellaceae*).

The abundance of *Deltaproteobacteria* rose over the course of composting from 4–6 to 6–8%. This tendency could be found in 10 of the 11 orders detected, *Bradymonadales* being the exception. However, abundance trends within the trials differed for the dominant order *Myxococcales* (E1: 5.4% constant; E2: 5.9% → 4.5%; E1-B: 4.1 → 4.5%; E2-B: 4.9 → 6.6%) and PB19 (max. 0.03%). *Myxococcales* were not only the most abundant, but also the most diverse order with 11 families, mostly consisting of only one affiliated genus.

Within the *Firmicutes*, *Bacilli* were the dominant group, showing a decline in relative abundance from start to end of composting (8–29% → 9–12%; with an exception in E2-B: 8% → 9%). The most abundant order was *Bacillales* with highest abundances in the 14-day samples, but an overall decline toward the end of composting (Figure 3). The relative abundance of *Bacillus*, however, rose from start to end of composting, correlating with the isolation study, where *Bacillus* spp. accounted for almost 37% of isolates.

The second most abundant order was *Lactobacillales*, which declined from 0.4 to 2.8% in the start samples to a maximum of 0.003% in the end samples. All other sequences within the class *Bacilli* belonged to uncultured representatives.

*Clostridia* were the second most abundant class within the phylum *Firmicutes*. Trends in relative abundance differed between compost piles, but numbers in general did not change much between start and end of composting (3–5% → 3–4%). Three orders were affiliated with *Clostridia*: *Clostridiales* being dominant with 2.5–4.8% of sequences in the start samples and 3.0–3.6% in the mature compost.

Relative abundance of 16S rRNA gene sequences of the phylum *Actinobacteria* did not change, but the occurrence of its dominant class *Actinobacteria* declined (12–17% → 6–7%), whereas other groups rose in numbers (*Acidimicrobiia* (0–1% → 7–9%), *Thermoleophilia*, *Nitriliruptoria*). The three most abundant orders within the class *Actinobacteria* were *Micrococcales*, *Streptosporangiales*, and *Corynebacteriales*, all of which declined during composting. *Corynebacteriaceae* with their only genus *Corynebacterium* showed increased numbers in the 14-day samples but disappeared at the end of composting.

**FIGURE 4:**
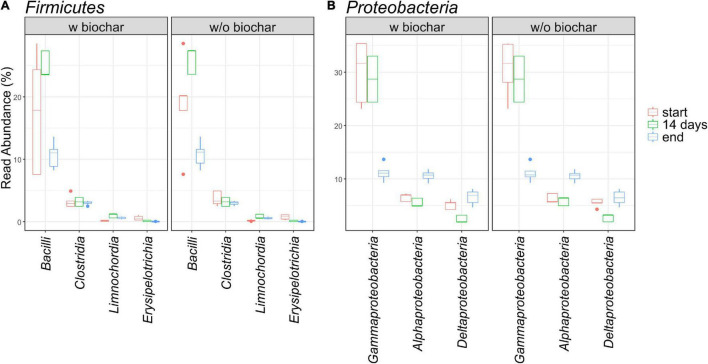
Relative read abundance of the affiliated classes within the phyla *Firmicutes* **(A)** and *Proteobacteria* **(B)** as depicted by boxplots.

The same pattern applied to *Bacteroidetes* with the number of *Bacteroidia* declining (7–14% → 7–9%) and the other two classes *Rhodothermia* (0–0.1% → 0.6–0.9%) and *Ignavibacteria* (0 → 0.1–0.5%) rising. The three most abundant orders within the *Bacteroidia* were *Cytophagales*, *Flavobacteriales*, and *Sphingobacteriales* of which the first one rose and the latter two declined over the course of composting. Interestingly, all three orders showed relative abundances in the 14-day samples opposing the general trend from start to end, meaning lowest levels in the *Cytophagales* 14-day samples and highest levels in the respective samples for *Flavobacteriales* (*Flavobacteriaceae*) and *Sphingobacteriales* (*Sphingobacteriaceae*). Besides, 14-day samples jumping out of the general trends were revealed for the order *Bacteroidetes*, as well as for orders *Corynebacteriales* and *Propionibacteriales* within the *Actinobacteria*, for *Clostridiales* within the *Firmicutes*, and within the *Proteobacteria* for *Rhizobiales* and *Sphingomonadales* (*Alphaproteobacteria*), *Myxococcales* (*Deltaproteobacteria*), *Cellvibrionales*, *Oceanospirillales*, and *Pseudomonadales* (*Gammaproteobacteria*). This list is based on individual observation of E1 and E1-B with averaged end samples (without E2 and E2-B), since 14-day samples were only available for repetition E1/E1B. When samples of each treatment were further averaged with start and end samples of E2 and E2-B, this pattern applies to more groups, including *Bacillales* and *Bacilli* within *Bacillales* (Figures 3, 4).

Among the most abundant phyla, *Chloroflexi* showed the highest richness with eight different classes (plus uncultured representatives). Numbers rose strongly from start to end of composting (1–5% → 13–15%). This trend was seen in all classes, especially in *Anaerolineae* (0–1% → 9–11%) and *Chloroflexia* (1–4% → 3–4%).

All classes and orders within *Gemmatimonadetes* and *Planctomycetes* showed a rising tendency from start to end of composting. Both groups held many uncultured representatives and a relatively low diversity at higher taxonomic resolution.

Differences in terms of relative abundances in the starting material from repetition 1 (E1 and E1-B) and repetition 2 (E2 and E2-B) were often observed. The repetitions were set up independently 2 weeks apart from each other. The averaged end samples, however, are usually very similar regardless of the starting relative abundance. Most of the outliers could be seen in only one of the four compost piles (E2-B) and in most of the cases in one of the biochar duplicates (E1-B, E2-B).

**FIGURE 5:**
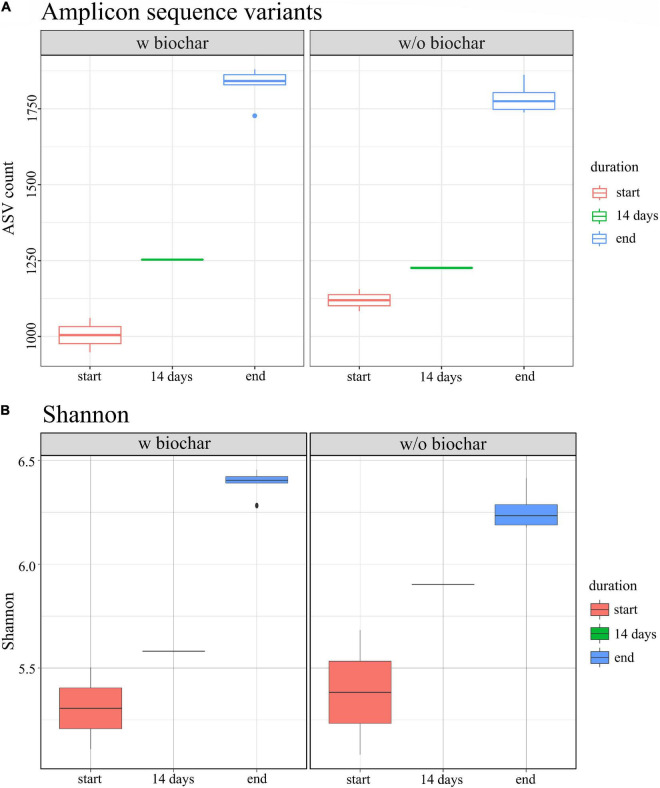
Alpha diversity of 16S rRNA gene amplicons for start (red), 14 days (green), and end samples (blue), based on **(A)** the number of amplicon sequence variants (ASV) and **(B)** Shannon indices.

Biochar treatment did neither show different composition or abundance of species compared with the non-biochar treatment, nor different tendencies of decrease or increase over composting. However, the alpha diversity was slightly higher as compared to the non-biochar samples. Nevertheless, the strongest change in diversity was observed between start and end samples of the composting process (Figure 5).

### Abundance of Antibiotic Resistance Genes Declines Over the Course of Composting

To get an insight into the number of different ARGs, their presence or absence in the compost, differences between non-biochar and biochar treatments, as well as the development and/or dissemination of resistance genes in the composting process, gene-specific PCRs were conducted.

Resistance genes against gentamicin [*aac6-aph2a, aph(2)-Ic*], erythromycin (*ermA*, *ermG*), sulfamethoxazole (*sul1, sul2*), and tetracycline (*tetL*) were detected in all samples. Both kanamycin resistance genes tested, as well as *ermB* and tetracycline resistance genes *tetB, tetK, tetM*, and *tetS*, were present in the start and partly the 14-day composting samples, but not in the end samples. *tetA* was present in starting and 14-days samples and in 1 out of 12 samples from the mature compost. *ampC, aph(2)-Ib*, *blaZ*, *ermC, ermD, mecA, vanB, qnrA, qnrB*, and *vanC1/C2* genes, chloramphenicol resistance genes *cat*_*pIP*501_, and *catLM*, as well as carbapenemase-genes *blaIMP, blaKPC, blaNDM-1, blaOXA-48*, and *blaCTX-M-15* were not present in any of the samples. No clear differences between biochar and non-biochar treatments were observed. The number of different resistance genes detected decreased from 15 in the starting samples to 8 in the mature compost (Table 1).

**TABLE 1:** Presence (+) and absence (−) of ARGs as assessed by PCR in start, 14 days, and end samples (front, middle, and back replicates of each pile) of composting.

Antibiotic	Resistance gene	E1 (start)	E1 (14 d)	E1 (front)	E1 (middle)	E1 (back)	E1-B (start)	E1-B (14 d)	E1-B (front)	E1-B (middle)	E1-B (back)	E2 (start)	E2 (front)	E2 (middle)	E2 (back)	E2-B (start)	E2-B (front)	E2-B (middle)	E2-B (back)	Start		14 d		End	
																				No biochar	biochar	No biochar	Biochar	No biochar	Biochar
Chloramphenicol	*cat*_*pIP*501_	−	−	−	−	−	−	−	−	−	−	−	−	−	−	−	−	−	−	−	−	−	−	−	−
	*catLM*	−	−	−	−	−	−	−	−	−	−	−	−	−	−	−	−	−	−	−	−	−	−	−	−
Fluoroquinolones	*qnrA*	−	−	−	−	−	−	−	−	−	−	−	−	−	−	−	−	−	−	−	−	−	−	−	−
	*qnrB*	−	−	−	−	−	−	−	−	−	−	−	−	−	−	−	−	−	−	−	−	−	−	−	−
Gentamicin	*aac6-aph2a*	+	+	+	+	+	+	+	+	−	+	+	+	−	−	−	−	−	−	+	+	+	+	+	+
	*aph(2)-Ib*	−	−	−	−	−	−	−	−	−	−	−	−	−	−	−	−	−	−	−	−	−	−	−	−
	*aph(2)-Ic*	+	+	+	+	−	+	+	+	−	+	+	+	+	+	+	+	+	+	+	+	+	+	+	+
Kanamycin	*aadD*_*pSK*41_	+	+	−	−	−	+	+	−	−	−	+	−	−	−	+	−	−	−	+	+	+	+	−	−
	*aph3-III*	+	+	−	−	−	+	+	−	−	−	+	−	−	−	+	−	−	−	+	+	+	+	−	−
β-lactams	*ampC*	−	−	−	−	−	−	−	−	−	−	−	−	−	−	−	−	−	−	−	−	−	−	−	−
	*blaZ*	−	−	−	−	−	−	−	−	−	−	−	−	−	−	−	−	−	−	−	−	−	−	−	−
	*blaIMP*	−	−	−	−	−	−	−	−	−	−	−	−	−	−	−	−	−	−	−	−	−	−	−	−
	*blaKPC*	−	−	−	−	−	−	−	−	−	−	−	−	−	−	−	−	−	−	−	−	−	−	−	−
	*blaNDM-1*	−	−	−	−	−	−	−	−	−	−	−	−	−	−	−	−	−	−	−	−	−	−	−	−
	*blaOXA-48*	−	−	−	−	−	−	−	−	−	−	−	−	−	−	−	−	−	−	−	−	−	−	−	−
	*blaCTX-M-15*	−	−	−	−	−	−	−	−	−	−	−	−	−	−	−	−	−	−	−	−	−	−	−	−
	*mecA*	−	−	−	−	−	−	−	−	−	−	−	−	−	−	−	−	−	−	−	−	−	−	−	−
Erythromycin	*ermA*	+	+	+	+	+	+	+	+	+	+	+	+	+	+	+	+	+	+	+	+	+	+	+	+
	*ermB*	+	+	−	−	−	+	+	−	−	−	+	−	−	−	−	−	−	−	+	+	+	+	−	−
	*ermC*	−	−	−	−	−	−	−	−	−	−	−	−	−	−	−	−	−	−	−	−	−	−	−	−
	*ermD*	−	−	−	−	−	−	−	−	−	−	−	−	−	−	−	−	−	−	−	−	−	−	−	−
	*ermG*	+	+	−	+	+	−	+	+	−	+	+	+	+	+	+	+	+	+	+	+	+	+	+	+
Sulfamethoxazole	*sul1*	+	+	+	+	+	+	+	+	+	+	+	+	+	+	+	+	+	+	+	+	+	+	+	+
	*sul2*	+	+	+	+	+	+	+	+	−	+	+	+	+	+	+	+	+	+	+	+	+	+	+	+
Tetracycline	*tetA*	+	+	−	−	−	+	+	−	−	−	+	−	−	+	+	−	−	−	+	+	+	+	+	−
	*tetB*	+	+	−	−	−	+	−	−	−	−	+	−	−	−	+	−	−	−	+	+	+	−	−	−
	*tetK*	+	+	−	−	−	+	+	−	−	−	−	−	−	−	−	−	−	−	+	+	+	+	−	−
	*tetL*	+	−	+	+	−	+	+	+	+	−	+	+	+	+	+	+	+	+	+	+	−	+	+	+
	*tetM*	+	−	−	−	−	+	+	−	−	−	+	−	−	−	+	−	−	−	+	+	−	+	−	−
	*tetS*	+	+	−	−	−	+	+	−	−	−	−	−	−	−	−	−	−	−	+	+	+	+	−	−
Vancomycin	*vanB*	−	−	−	−	−	−	−	−	−	−	−	−	−	−	−	−	−	−	−	−	−	−	−	−
	*vanC1/C2*	−	−	−	−	−	−	−	−	−	−	−	−	−	−	−	−	−	−	−	−	−	−	−	−

*E1 and E1-B, first repetition, E2 and E2-B, second repetition, “−B” indicating biochar treatment. The last columns (black frame) show the results from the respective replicates combined by time-point [start, 14 days (no replicates available), end], as well as by treatment (no biochar, biochar). “+” indicates that at least one of the replicates was positive.*

## Discussion

The present study aimed to assess the hygienization success of a thermophilic composting trial of dry toilet contents. We used cultivation-based, as well as molecular approaches, to analyze both the occurrence of human-pathogenic bacteria in the mature compost and the fate of antibiotic resistance genes over the process of composting.

### Assessment of Fecal Contamination

*Escherichia coli* MPN as indicator for fecal contamination showed levels consistent with the limits of 1 x 10^3^ CFU/g of treated sewage sludge suggested by the German Federal Ministry for the Environment, Nature Conservation, and Nuclear Safety (Pietsch et al., 2014) and European Commission (Carrington, 2001). *Salmonella* MPN showed elevated levels in one compost pile (E1) due to one positive result in one of the triplicates analyzed. Therefore, the requirement of most German regulations of 0 CFU/50 g of fertilizer was not met for pile E1 (Bioabfallverordnung, 1998; Düngemittelverordnung, 2012; Klärschlammverordnung, 2017). In the other three piles E1-B, E2, and E2-B, *Salmonella* was not detected. Thus, they fulfill the criteria for fertilizers.

Germer et al. (2010) assessed the hygienization efficiency of an open composting trial of fecal matter and shredded plant waste using cultivation-based methods to detect different indicator organisms. In all samples of the mature compost, *Salmonella* was found with about 3 x 10^4^ CFU/g. Poor temperature development during the trial was discussed as a possible cause, which in turn was linked to the lack of easily accessible organic compounds for the composting process to start. This also applies to the present trial with main carbon sources from ligneous material (sawdust and plant waste) that are not easily degradable. Therefore, temperatures remained rather low and did not match the German regulations for compost [minimum temperatures of 55°C for 2 weeks, 60°C for 6 days, or 65°C for 3 days, Bioabfallverordnung (1998)]. In a conclusive study by Ceustermans et al. (2007), the influence of temperature was highlighted as the most important variable affecting the survival of *Salmonella enterica* ssp. *enterica* serotype Senftenberg during composting. Besides, moisture content and microbial competition were found to influence survival of *Salmonella*. The authors identified a combination of 60°C and 60–65% moisture during composting as sufficient to inactivate *Salmonella* within 10 h (Ceustermans et al., 2007). In the present study, temperatures in piles without biochar only reached 60°C in the surface area, but not in the compost center, and biochar composts did not reach 60°C at all (data not shown). In all piles, surface temperatures exceeded temperatures in the compost center, which indicates that aeration and therefore oxygen supply was insufficient. Initial moisture matched Ceustermans’ criteria for the first repetition (E1 and E1-B), but not the second, where moisture exceeded 65%. Too high moisture contents limit the oxygen supply (Godlewska et al., 2017), which would explain the poor temperature development in the second repetition. In pile E1, however, where *Salmonella* was detected, moisture ranged between 48 and 63% during the composting process (data not shown). In general, moisture contents between 50 and 60% are considered to be optimal (Bernal et al., 2009), but according to Ceustermans’ finding neither temperature nor moisture was sufficient for *Salmonella* removal. Hence, composting parameters need to be further evaluated to ensure sufficient pathogen removal.

### Opportunistic Human Pathogens Account for 36.5% of Isolated Bacteria From Mature Compost Due to the Selective Culture Conditions

The isolation study was conducted to evaluate the load of culturable potential human pathogenic bacteria in the mature compost, with an additional focus on antibiotic resistant pathogens. *Bacillus* and *Brucella* were found to be the most abundant genera within the isolates, followed by *Serratia* and *Pseudomonas*.

Of the targeted species, only *B. cereus* and *P. aeruginosa* were identified, whereas *Acinetobacter* or *Enterococcus* spp. could not be isolated. Moreover, only two Staphylococci were found, and none of them was identified as the serious and often multiple resistant nosocomial pathogen *S. aureus*.

*Bacillus* spp. are ubiquitous and found in habitats, such as soil and sediments, dust, plants, and the rhizosphere, where they promote plant growth and inhibit plant pathogens. They were isolated from stool samples and the gut of healthy persons, but they can also cause food poisoning due to their ability to produce toxins. *B. cereus*, which has been isolated in this study (Supplementary Table 10), is known as an opportunistic pathogen. The ability to form spores helps to resist unfavorable conditions and contaminate, for example, food products (Arnesen et al., 2008; Mongkolthanaruk, 2012; De-la-Peña and Loyola-Vargas, 2014; Ehling-Schulz et al., 2019). The *B. cereus* isolate of this study (E1-B) shows (intermediate) resistance to ampicillin, sulfamethoxazole, and vancomycin. Clinical strains are often resistant to beta-lactam antibiotics, which is in line with the ampicillin resistance in this isolate (Ehling-Schulz et al., 2019). Trimethoprim/sulfamethoxazole resistance has been described in some isolates before, whereas vancomycin resistance seems rather unusual (Luna et al., 2007; Bottone, 2010; Sun et al., 2020).

The second largest group of isolates belongs to the species *B. intermedia* (isolated from all piles, Supplementary Table 10), formerly known as *O. intermedium* (Velasco et al., 1998; Hördt et al., 2020). The species has been isolated from xenobiotic-polluted environments, such as chromium- or lead-polluted soil (Waranusantigul et al., 2011; Kavita and Keharia, 2012), oilfields (Chai et al., 2015), as well as tobacco waste (Yuan et al., 2005). On the other hand, *Brucella* species cause the common zoonotic infection brucellosis (Godfroid et al., 2011). *B. intermedia* is reported as a rare opportunistic pathogen (Möller et al., 1999; Apisarnthanarak et al., 2005; Bharucha et al., 2017; Kassab et al., 2021). Clinical isolates usually express high antibiotic resistance levels (Teyssier et al., 2005; Thoma et al., 2009). This tendency does not match our results with only a few multiple resistant *B. intermedia* isolates. Since resistance breakpoints for agar diffusion assays for this genus were not accessible, we tested only four antibiotics, possibly missing the full antibiotic resistance potential of these *B. intermedia* isolates.

*Serratia* spp. (isolated from E1, E1-B, and E2-B, Supplementary Table 10) also cover a wide range of habitats, including water, soil, plants and rhizosphere, insects, and animals, either in a harmless or a pathogenic way. *S. marcescens*, one of the most prevalently identified isolates in the present study (E1, E1-B, and E2-B, Supplementary Table 10), has been revealed as a natural member of the bacterial community of different soil types. On the other hand, *Serratia* is pathogenic to humans and one common cause for bloodstream infections and pneumonia. Especially *S. marcescens* is a nosocomial and opportunistic human pathogen. Clinical isolates usually show high levels of resistance. Multiple resistant strains are frequently found, also due to intrinsic resistance to several antibiotics (Mahlen, 2011). It is therefore not surprising that some of the isolates in the present study show multiple resistances. One could speculate that the comparatively low resistance levels of other *S. marcescens* isolates indicate an environmental rather than human-associated origin.

All *S. maltophilia* isolates from the present work (E2 and E1-B, Supplementary Table 10) exhibit resistance to many of the tested antibiotics. Resistance to, for example, aminoglycosides, beta-lactam antibiotics, fluoroquinolones, macrolides, tetracyclines, and trimethoprim-sulfamethoxazole is well known in *S. maltophilia* and the mechanisms have been extensively studied. Intrinsic resistance is high due to low membrane permeability, chromosomal genes encoding efflux pumps, beta-lactamases, and antibiotic-modifying enzymes. Additionally, horizontal gene transfer can increase the resistance level. The species is involved in respiratory tract infections, but has also been isolated from environmental sources like soil, the rhizosphere, or aquatic habitats [reviewed by Brooke (2012)].

Regarding the isolation study, no concrete differences in diversity could be observed. Most genera were slightly more abundant in the non-biochar treatment. The species variety is almost equal, especially when taking into consideration that more isolates were obtained from the non-biochar treatment (52%). However, one notable difference is the occurrence of multiple resistant isolates, of which two-thirds originate from the biochar treatments. Interestingly, no differences between the treatments have been detected in terms of the molecular analysis of antibiotic resistance genes in compost DNA extracts. Different biochar types, as well as the combination of biochar type with different manures, were shown to influence the behavior of ARGs over composting (Cui et al., 2017). Biochar was found beneficial for the composting process due to better aeration and the porous structure of biochar serving as a habitat for microbes (Sanchez-Monedero et al., 2018). It is therefore possible, that the higher number of multiresistant isolates in the biochar treatments is due to additional niches for their survival. Another explanation could be co-selection of antibiotic and heavy metal resistance genes. While other metals analyzed showed a higher concentration in the compost without biochar, the amount of copper was higher in the biochar treatments as compared with the control (data not shown). Copper and antibiotic resistance genes are often located on the same plasmid and plasmids encoding both metal resistance genes and ARGs are predominantly conjugative (Baker-Austin et al., 2006; Pal et al., 2015). Coincidently, the presence of copper was shown to promote conjugative transfer of plasmids encoding multiple resistances, and copper to have greater impact than other heavy metals on conjugative transfer events (Zhang et al., 2018, 2019). A positive correlation of decreases of ARGs and heavy metal concentration was also reported for chicken manure and sewage sludge compost (Cui et al., 2016; Qiu et al., 2021). Therefore, the presence of the metal likely maintained selective pressure for the benefit of antibiotic resistant bacteria.

Although more than one third of the isolated bacteria belong to BSL-2, only a few serious human pathogens targeted by the isolation strategy, were identified (*B. cereus* and *P. aeruginosa*), and other BSL-2 isolates were present only in small numbers. The identified species also occur naturally in soil environments and are known as opportunistic rather than strict pathogens. Of BSL-2 isolates, 79% exhibited no or only few resistances. All these findings point to a hygienization of the substrates over the course of composting.

### Shift of the Bacterial Community Over Composting Toward Soil Associated Phyla

16S rRNA gene amplicon analysis identified *Proteobacteria*, *Firmicutes*, *Actinobacteria*, *Chloroflexi*, *Bacteroidetes*, *Gemmatimonadetes*, and *Planctomycetes* as the most abundant phyla in starting material and mature compost of human feces from dry toilets. Throughout composting trials from different feedstocks and composting scales and methods, *Firmicutes* and *Proteobacteria* have been demonstrated to be the predominant groups throughout the process, along with *Actinobacteria*, *Bacteroidetes*, or *Planctomycetes* (Esperón et al., 2020). Our results are in accordance with other manure and sewage composting experiments revealing the same predominant phyla (*Proteobacteria*, *Firmicutes*, *Actinobacteria*, and *Bacteroidetes*), although relative abundances of the respective phyla vary across experiments and treatments. In many studies, relative abundance of *Proteobacteria* in the mature compost varies between around 10 and 30% (Wang et al., 2016; Guo et al., 2017; Lu et al., 2018; Duan et al., 2019). However, exceptions have been reported, as well: Shi et al. (2018) found abundances of about 40–50% of *Proteobacteria* in the mature compost, although a comparatively low relative abundance was detected in the compost feedstock. Huge differences have been reported for *Firmicutes*’ relative abundances in mature compost, ranging from maxima of 5% (Shi et al., 2018) to around 60% (Wang et al., 2016). Duan et al. (2019) revealed *Actinobacteria* as the most abundant phylum in mature cattle manure compost with abundances between 30 and 40% of the total bacterial community. In contrast, Guo et al. (2017) and Lu et al. (2018) reported *Actinobacteria* abundance of 10–20% and Wang et al. (2016) and Shi et al. (2018) up to 5% (Wang et al., 2016; Shi et al., 2018). Similar differences can be found for *Bacteroidetes* relative abundance in the mature compost with less than 3% (Guo et al., 2017), around 10% (Wang et al., 2016; Lu et al., 2018), 20–30% (Duan et al., 2019), and approximately 40% (Shi et al., 2018). The trials differed with regard to the substrates: cattle manure, wheat stalks and inoculated *B. subtilis* (Duan et al., 2019), swine manure, wheat straw and sodium polyacrylate (Guo et al., 2017), swine manure, wheat straw and coal gasification slag (Lu et al., 2018), swine manure, mushroom residues and red mud (Wang et al., 2016), human feces, and sawdust and tetracycline at different concentrations (Shi et al., 2018). Wang et al. (2016) conducted windrow composting, whereas the other authors used containers of different sizes, mostly passively aerated, with the exception of Shi et al. (2018). All trials conducted turning of the compost, but with different frequencies, and composting duration also varied from 21–52 days between experiments. Since the parameters of composting were extremely diverse, so might be the conditions in the compost in terms of aeration, composition of nutrients or toxic substances, accessible carbon and nitrogen, and competing microbial species. It is therefore hard to identify specific reasons for the differences observed in the bacterial community. Certain phyla usually occur in compost, but the proportions are different with different feedstocks and composting methods and consequently varying physico-chemical parameters.

The number of *Proteobacteria* was strongly reduced from start to end of composting in the present study. This is in accordance with Duan et al. (2019), who reported reductions in the relative abundance of the phylum during cattle manure composting to around 15–30% in the mature compost. In other composting experiments of manure and sewage sludge, *Proteobacteria* increased from rather low levels to a relative abundance of around 20–30% in the mature compost (Guo et al., 2017; Lu et al., 2018). This may indicate that the bacterial community is more similar after than before composting, independent of the substrates composted, an idea already suggested by Franke-Whittle et al. (2009).

Significant declines of *Gammaproteobacteria* during manure composting and decline of *Pseudomonas* as its most abundant genus have been described before, which corresponds to the results of our study (de Gannes et al., 2013; Zhong et al., 2018). *Pseudomonas* species are very diverse and comprise degraders of various organic compounds, as well as nitrogen-fixing species or plant- and human-pathogenic bacteria (Kersters et al., 2006). It is therefore not surprising to find this genus throughout composting. Abundances declined strongly toward the end of composting, suggesting that the majority belonged to the gastro-intestinal microbiome that did not survive the composting conditions. The same applies to the genus *Acinetobacter* that peaked after 14 days of composting but almost disappeared in the mature compost. The genus is associated with nosocomial pathogens, as well as with the autochthonous soil microbiome (Kersters et al., 2006).

de Gannes et al. (2013) described *Gammaproteobacteria* to be very abundant in the starting phase of composting, but abundances of *Gamma*- and *Alphaproteobacteria* converge during the thermophilic and maturation phase. This was detected in our study, as well, with *Gammaproteobacteria* declining from 23–35 to 11–12% and *Alphaproteobacteria* rising from 6–7 to 10–11% relative abundance in the mature compost. The phylum *Alphaproteobacteria* comprises a variety of members, for example *Acetobacter* species that are utilized for vinegar production, as well as the plant tumor-inducing *Agrobacterium* or nitrogen-fixing plant-symbiont *Rhizobium* (Kersters et al., 2006). Since a pool of functions facilitates adaptation to changing conditions and many plant-associated members are adapted to soil environments, it is not surprising to find *Alphaproteobacteria* and the order *Rhizobiales* rising in relative abundance over composting. The most abundant genera within the *Alphaproteobacteria* described by de Gannes et al. (2013) correspond to the most abundant families found in the present study.

*Firmicutes* are found in diverse environments and are one of the most abundant phyla commonly found in soils (Govil et al., 2021). The authors found the class *Bacilli* dominating in yard and municipal solid waste compost samples, followed by *Clostridia*, which is in concordance with the present study. Here, the class *Bacilli* and the order *Bacillales* were the dominant members of the *Firmicutes* with strong peaks in relative abundance in the 14-day samples. Members of this genus are important for decomposing organic matter and dissolved organic carbon and can withstand a broad range of pH and temperatures (Jain et al., 2012; Liu et al., 2018).

Interestingly, in the present study the number of *Chloroflexi* was found to increase strongly from start to end of composting to one of the most abundant phyla in the mature compost. An increase of relative abundance of *Chloroflexi* in the cooling and maturation phase has been recently described for cattle manure compost (Bello et al., 2020). Prominent increases from zero clones in the beginning to 10% of clones at the end have been shown in a clone library from dairy manure compost by Tian et al. (2013), as well.

Summarizing, our study revealed patterns of de- or increases of bacterial groups that have been described for manure or sewage sludge compost before. It was shown that the bacterial community was strongly reshaped as a result of the composting process with clades decreasing that comprise well-known pathogens. It can therefore be assumed that the pathogenic potential of the initial material was reduced during composting.

Alpha diversity increased over composting in the present study. Rising alpha diversity over composting has been reported for manure compost before (de Gannes et al., 2013; Bello et al., 2020). One driver of microbial succession is environmental stress such as the rising temperatures during composting (Fierer et al., 2010). Compost alpha diversity was found to be most strongly influenced by the composting phase, whereas compost types did not show any effects (de Gannes et al., 2013; Zainudin et al., 2017; Bello et al., 2020). Differences in diversity over composting might, however, be due to differences in the feedstock composition, which could affect how the composting progresses (Klammer et al., 2008).

Moreover, slightly higher alpha diversity was detected in the biochar treatments compared to the treatments without biochar. Similar findings have been reported previously (Wei et al., 2014; Bello et al., 2020; Yan et al., 2021). Reasons for increased diversity could be the large surface and porous structure of biochar, where bacteria find additional habitats. Further effects comprise increased water and nutrient retention, the absorption of toxins, improved aeration, changes in pH and *C*:*N* ratio, which directly or indirectly influence the bacterial community. However, the effects are also due to an interplay with management strategies, for example, in piles under regular irrigation, biochar increases the water retention capacity and prevents drying out, whereas it increases drying in non-irrigated composts (Sanchez-Monedero et al., 2018). This might explain differences in results on biochar composting and underlines the importance of evaluating suitable combinations of composting parameters to achieve a functioning composting process and beneficial effects of the supplements.

Bacterial community structure may possibly be biased due to the sample preparation prior to DNA extraction. Since the starting material before composting was bulky, it had to be homogenized before analysis. Thus, the samples were dried at 40°C and ground. This drying period might have caused changes in the relative abundances of the bacterial clades, since the temperature can generate selective pressure and adaptation to desiccation differs between species (Bosch et al., 2021). It is, however, difficult to predict changes in a complex environment like compost or soil. Reactions of bacteria are dependent on physicochemical parameters, like moisture, pH, or *C*/*N* ratio, for example, and their interplay (Ahn and Peralta, 2009; Chodak et al., 2015). Although compost differs from soil and soils are highly diverse, studies on the effect of soil drying on the microbial community can give insight into the possible effects of drying compost samples. The effect of preserving soil by 2 weeks air-drying at ambient temperatures and subsequent storage at room temperature for several months on molecular analysis of the bacterial community was compared with DNA extracted from the respective fresh material. The authors found reduced DNA yields and minimal changes of species richness and bacterial community structure in the preserved soils as assessed by 16S rRNA gene DGGE (Campbell et al., 2009). The effect of different storage methods, including air-drying, was found to exhibit minor effects on the microbial soil profiles as assessed by using PCR and capillary electrophoresis single-strand conformation polymorphism (Larson, 2012). Air-drying of soil at 42°C for 48 h was found to influence 16S rRNA gene DGGE results less than the environmental parameters of the soils compared, like, for example, pH, soil, or fertilizer type (Tzeneva et al., 2009). Use of fresh material will draw the most reliable picture of the bacterial community and should be preferred over dried samples, whenever possible. Nevertheless, it can be assumed that samples with the same pre-treatment are comparable among each other and differences within these samples are reliable.

### Composting Decreases the Diversity of PCR Tested Antibiotic Resistance Genes by 50%

PCR analysis of the occurrence of ARGs showed seven resistance genes less in the mature compost than in the start material. The tetracycline resistance genes *tetB, tetK, tetM*, and *tetS*, the kanamycin resistance genes *aadD*_*pSK*41_, *aph3-III*, and the erythromycin resistance gene *ermB* were not detectable in the mature compost. In addition, the *tetA* resistance gene was only present in the start and one of the mature compost samples, indicating decreased abundance over composting, as well.

ARG reduction is in general most strongly influenced by temperature, which kills non-thermophilic bacteria harboring ARGs, but mechanisms are not well understood (Pereira et al., 2021). ARG persistence is likely due to horizontal gene transfer to heat-resistant species (Enne et al., 2001; Sköld, 2001; Pereira et al., 2021). Besides, the microbial community affects the fate of ARGs, since different genera are associated with different genes and the community changes strongly over the composting process. Especially genera within *Firmicutes* and *Proteobacteria* are likely to host ARGs, and both phyla declined over composting in the present study. Differences in the microbial community are in turn connected with changes in abiotic factors, like temperature, pH, or moisture. Selective pressure through antibiotics or residues may also influence the persistence of ARGs. Besides, co-selection through heavy metals was found to influence ARG removal, since antibiotic and metal resistance genes are often located on the same MGE (Ezugworie et al., 2021; Liu et al., 2021; Pereira et al., 2021).

Different works report a decline in abundance of various tetracycline resistance genes over manure composting (Wang et al., 2016; Cheng et al., 2019; Esperón et al., 2020). This agrees with the decrease of *tetA*, *tetB, tetK, tetM*, and *tetS* genes in our study, which could be detected in the starting material, but not in the mature compost. Contrary to this, *tetL* was also detected after composting. The persistence of the *tetL* gene over composting has been already described by Xie et al. (2016) for manure composting. Reasons for this could be the functional diversity of the encoded antiporter that plays a role in the response to sodium chloride and alkali stresses, as well as potassium insufficiency (Krulwich et al., 2001). Moreover, the gene is associated (not exclusively) with *Bacillus* spp. which were found highly abundant in the mature compost as assessed by both the isolation study and 16S rRNA gene amplicon sequencing. Since *tetL* is usually encoded on plasmids, it is possible that the gene was spread *via* horizontal gene transfer to heat-resistant bacteria before the thermophilic phase, resulting in detectable levels of the gene in the mature compost (Kadlec et al., 2012).

Like in our study, the abundance of the *ermB* resistance gene, along with other macrolide resistance genes, has been shown to decline over composting (Li et al., 2017; Esperón et al., 2020). *ermB* is often located on the same conjugative transposons in Streptococci like *tetM*, which was likewise not detected in the mature compost (Varaldo et al., 2009). *ermA* and *ermC* declined over composting of different animal manure in a study of Zhou and Yao (2020), whereas *ermA* was present in all samples in our experiment and the latter was not detectable at all. These differences could likely be attributed to the higher sensitivity of the method used by Zhou and Yao (2020). *ermA* is usually found on transposons, *ermC* on small plasmids of Gram-positive bacteria, which would allow for the transfer of the genes to other bacteria before the thermophilic phase (Varaldo et al., 2009; Kadlec et al., 2012; Sarrou et al., 2019).

Sulfonamide resistance genes showed persisting –or more often even increasing– levels over animal manure composting in several studies (Guo et al., 2017; Li et al., 2017; Lu et al., 2018; Cheng et al., 2019; Duan et al., 2019; Esperón et al., 2020). Despite a decline in sulfonamide resistance gene numbers during hyperthermophilic composting of sewage sludge, numbers rose again during the cooling phase (Liao et al., 2018). This agrees with our findings: both *sul1* and *sul2* genes were present in all samples. *Sul1* is often found on class 1 integrons and *sul2* on small plasmids in Gram-negative bacteria. The genes have been found highly transferable *via* conjugation (Sköld, 2001; Jiang et al., 2019).

In our study no beta-lactam ARGs were detected in any sample. In an extensive study on ARGs before and after manure composting, various beta-lactam ARGs were analyzed, including *ampC*, *blaCTX-M*, *blaOXA*, *blaZ*, and *mecA* genes. Most genes were generally low in abundance and decreased over composting, in many cases below the detection limit (Zhou and Yao, 2020).

Investigations on the effect of compost treatments similar to those of our study are scarce. Lu et al. (2018) suggested the addition of 10% coal gasification slag for better removal of ARGs in compost. In another study, positive effects of biochar on the removal of ARGs were described for 8 out of 12 ARGs analyzed (Li et al., 2017). In the present study, no ARG reducing effects of the biochar treatment could be observed. Besides, reduction dynamics of ARGs seem to depend on various factors, such as compost substrates (Qian et al., 2018). Joy et al. (2014) found differences in the persistence of different ARGs in animal manure. *Tet* genes were not detected after the degradation of the selective antibiotic chlortetracycline, whereas *ermB* was detectable without tylosine, suggesting that other compounds maintain selective pressure or selection is not necessary to sustain these ARGs.

The study showed the disappearance of half of the ARGs detected in the feedstock material indicating that composting can be effective in the hygienization of human excreta from dry toilets. Taken together, the results suggest that the mature compost does not pose a high risk of spreading clinical strains to the environment when further used in agriculture. However, further evaluation of the fate of pathogenic bacteria during field trials should be conducted.

To our knowledge, the present work is the first to assess ARGs in compost from dry toilet contents. Previous studies have only focused on the fate of pathogens (Tønner-Klank et al., 2007; Germer et al., 2010; Mehl et al., 2011; Sossou et al., 2014). In most studies on manure or sewage sludge composting regarding the fate of ARGs, laboratory-scale experiments were conducted instead of large-scale composting in windrows. Moreover, composting periods were shorter in most studies (Ezzariai et al., 2018). Sewage sludge compost is most closely related to dry toilet contents. Composting was usually conducted on dewatered sludge, the material after the first stage of wastewater treatment (Ezzariai et al., 2018; Schnell et al., 2020). Studies of which on the fate of ARGs resulted in varying results (Zhang et al., 2016; Jang et al., 2018; Liao et al., 2018; Cui et al., 2020; Wei et al., 2020). In the present study we assessed a realistic composting scenario of dry toilet contents in terms of set-up and composting period, which provided insights into the fate of pathogens, ARGs, and changes in the bacterial community.

## Data Availability Statement

The datasets presented in this study can be found in online repositories. The names of the repository/repositories and accession number(s) can be found in the article/Supplementary Material.

## Author Contributions

EG and KW designed and supervised the laboratory experiments. KW, AP, DS, KE-S, MW, and IL performed the laboratory experiments and analyzed the data. KP, NB, and TH designed the composting trial. TH conducted process control. KW, DS, AP, TH, and EG wrote the manuscript and designed all figures and tables. RD contributed with insightful discussions on analysis and interpretation of the data. All authors interpreted the results, read, and revised the manuscript.

## Conflict of Interest

The authors declare that the research was conducted in the absence of any commercial or financial relationships that could be construed as a potential conflict of interest.

## Publisher’s Note

All claims expressed in this article are solely those of the authors and do not necessarily represent those of their affiliated organizations, or those of the publisher, the editors and the reviewers. Any product that may be evaluated in this article, or claim that may be made by its manufacturer, is not guaranteed or endorsed by the publisher.

## Footnotes

1
http://www.eucast.org
