# Supplementary material for: Thermophilic Composting of Human Feces: Development of Bacterial Community Composition and Antimicrobial Resistance Gene Pool
Source: Front Microbiol. 2022 Feb 18;13:824834. doi: 10.3389/fmicb.2022.824834 (PMC8895236; doi:10.3389/fmicb.2022.824834)
Supplement: Supplementary file 1 [file Data_Sheet_1.docx]

Table S1. Initial composition of composting substrates as percentage by volume and weight (calculated based on the density and the measured volumes of the component).

|  | **E1*** |  | **E1-B°** |  | **E2^+^** |  | **E2-B^#^** |  |
| --- | --- | --- | --- | --- | --- | --- | --- | --- |
|  | (v/v) | (w/w) | (v/v) | (w/w) | (v/v) | (w/w) | (v/v) | (w/w) |
| dry toilet contents | 62.6 % | 79.8 % | 60.6 % | 77.8 % | 63.0 % | 80.6 % | 60.4 % | 77.4 % |
| green cuttings | 31.3 % | 16.3 % | 30.3 % | 15.9 % | 31.2 % | 16.3 % | 30.8 % | 16.2 % |
| straw | 5.0 % | 1.4 % | 4.9 % | 1.4 % | 5.0 % | 1.4 % | 4.9 % | 1.4 % |
| urine | 1.1 % | 2.5 % | 0.5 % | 1.1 % | 0.7 % | 1.7 % | 0.9 % | 2.0 % |
| biochar | --- | --- | 3.7 % | 3.9 % | --- | --- | 3.0 % | 3.1 % |

* E1: repetition 1, treatment without biochar; ° E1-B: repetition 1, treatment with biochar; ^+^ E2: repetition 2, treatment without biochar; ^#^ E2-B: repetition 2, treatment with biochar

Table S2. Proportion of dry matter, volatile solids and C/N-ratio of the main compost substrates.

|  | **dry matter (DM)*** | **volatile solids (VS)^+^** | **C/N-ratio** |
| --- | --- | --- | --- |
| dry toilet contents | 25.4 ± 1.2 % | 83.4 ± 0.6 %DM | 35 ± 7 |
| green cuttings | 73.2 ± 1.6 % | 54.7 ± 6.2 %DM | 23 ± 1 |
| straw | 85.3 ± 2.5 % | 85.8 ± 13.4 %DM | 77 ± 14 |

* assessed through weight difference before and after drying at 105 °C for 24 h

+ assessed through weight difference before and after combustion at 550°C using dried samples (105 °C, 24 h)


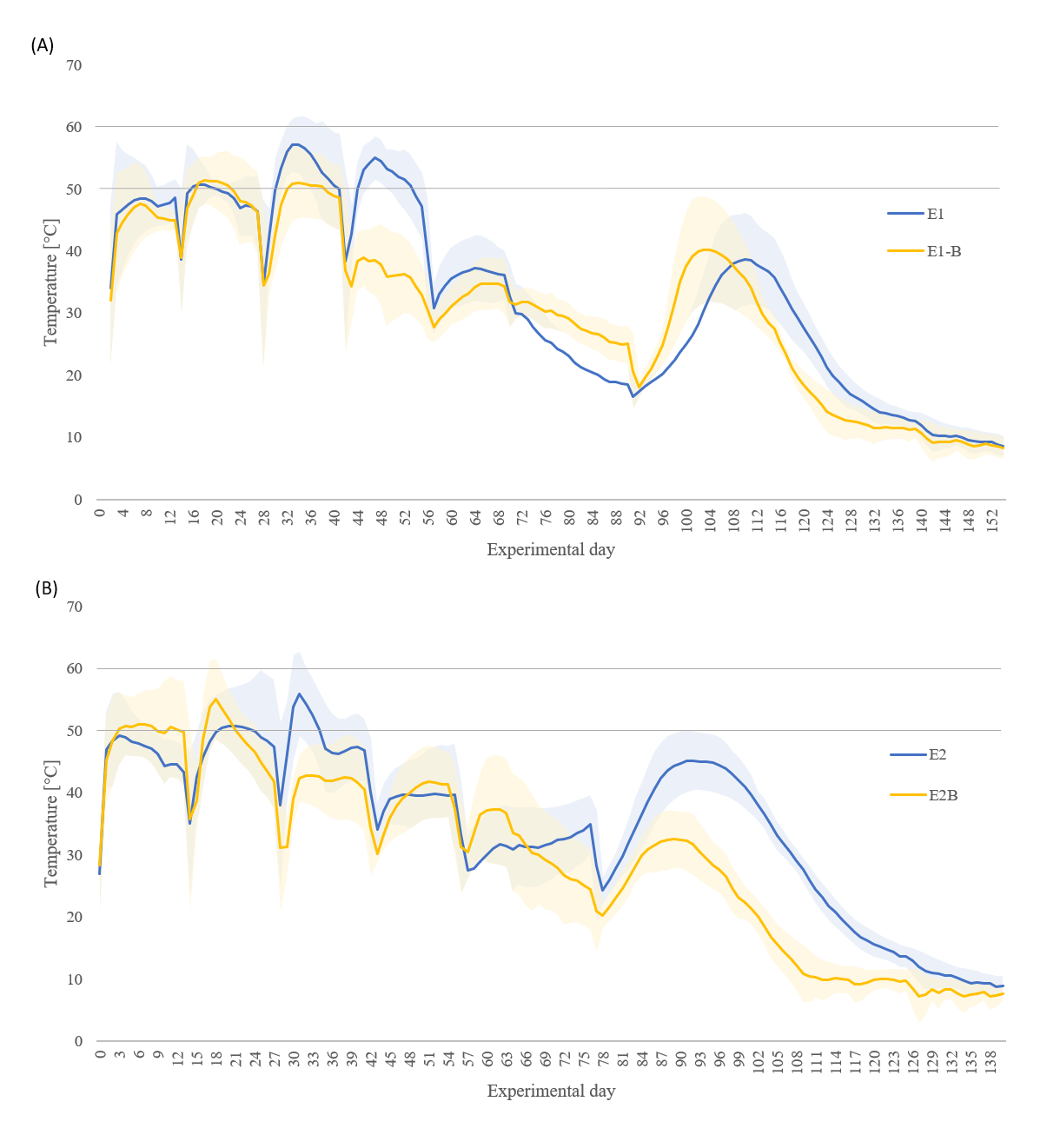


Figure S1. Average temperature and standard deviation for compost repetition 1 (A) and 2 (B). Blue color represents the treatments without biochar and yellow the treatments with biochar. Temperature was recorded every two hours at six positions in each compost pile (3 x compost center, 3 x surface area; front, middle, back position, respectively). Data represent the average of all measurements of the same date and all positions (72 individual measurements), with the exception of first and last days of temperature recording.

Table S3. Average temperature (temp.) and standard deviation for compost piles E1, E1-B, E2 and E2-B. Temperature was recorded every two hours at six positions in each compost pile (3 x compost center, 3 x surface area; front, middle, back position, respectively). Data represent the average of all measurements of the same date and all positions (72 individual measurements), except first and last days of temperature recording. E1/E1-B were set up on 15/08/2018, E2/E2-B on 29/08/2018.

| Date | E1 | | E1-B | | E2 | | E2-B | |
| --- | --- | --- | --- | --- | --- | --- | --- | --- |
|  | Average temp.[°C] | Standard deviation | Average temp. [°C] | Standard deviation | Average temp. [°C] | Standard deviation | Average temp. [°C] | Standard deviation |
| 17.08.2018 | 34.1 | 13.3 | 32.2 | 10.9 |  |  |  |  |
| 18.08.2018 | 45.9 | 11.6 | 42.9 | 9.7 |  |  |  |  |
| 19.08.2018 | 46.7 | 9.5 | 44.5 | 8.5 |  |  |  |  |
| 20.08.2018 | 47.5 | 8.1 | 45.9 | 7.8 |  |  |  |  |
| 21.08.2018 | 48.1 | 6.9 | 47.0 | 7.2 |  |  |  |  |
| 22.08.2018 | 48.4 | 6.0 | 47.6 | 6.4 |  |  |  |  |
| 23.08.2018 | 48.5 | 5.2 | 47.4 | 5.2 |  |  |  |  |
| 24.08.2018 | 48.0 | 4.2 | 46.4 | 3.6 |  |  |  |  |
| 25.08.2018 | 47.2 | 3.2 | 45.3 | 2.1 |  |  |  |  |
| 26.08.2018 | 47.4 | 3.2 | 45.2 | 1.9 |  |  |  |  |
| 27.08.2018 | 47.7 | 3.4 | 45.0 | 1.8 |  |  |  |  |
| 28.08.2018 | 48.6 | 3.1 | 45.0 | 1.6 |  |  |  |  |
| 29.08.2018 | 38.7 | 11.2 | 39.0 | 9.2 | 26.9 | 7.3 | 28.4 | 7.4 |
| 30.08.2018 | 49.3 | 7.9 | 46.9 | 6.5 | 47.0 | 6.1 | 45.3 | 7.1 |
| 31.08.2018 | 50.4 | 6.2 | 49.1 | 4.9 | 48.4 | 7.6 | 48.4 | 7.1 |
| 01.09.2018 | 50.8 | 4.3 | 51.0 | 4.0 | 49.1 | 7.2 | 50.4 | 5.8 |
| 02.09.2018 | 50.7 | 2.8 | 51.4 | 3.8 | 48.8 | 6.0 | 50.8 | 4.8 |
| 03.09.2018 | 50.3 | 1.6 | 51.3 | 3.9 | 48.2 | 4.8 | 50.7 | 4.7 |
| 04.09.2018 | 50.0 | 1.0 | 51.3 | 4.5 | 47.9 | 4.2 | 51.0 | 5.1 |
| 05.09.2018 | 49.6 | 1.2 | 51.0 | 5.0 | 47.5 | 3.6 | 51.0 | 5.4 |
| 06.09.2018 | 49.3 | 1.7 | 50.6 | 5.4 | 47.1 | 3.1 | 50.7 | 5.9 |
| 07.09.2018 | 48.5 | 2.7 | 49.5 | 5.8 | 46.2 | 3.2 | 50.0 | 6.9 |
| 08.09.2018 | 46.9 | 4.6 | 48.0 | 7.1 | 44.3 | 4.4 | 49.7 | 8.3 |
| 09.09.2018 | 47.3 | 4.8 | 47.9 | 6.6 | 44.6 | 4.1 | 50.6 | 8.1 |
| 10.09.2018 | 47.3 | 4.7 | 47.4 | 6.0 | 44.6 | 3.5 | 50.2 | 7.9 |
| 11.09.2018 | 46.3 | 5.8 | 46.2 | 6.7 | 43.2 | 4.5 | 49.7 | 8.4 |
| 12.09.2018 | 34.7 | 13.6 | 34.5 | 13.5 | 35.1 | 11.6 | 35.7 | 14.7 |
| 13.09.2018 | 42.1 | 4.8 | 36.3 | 3.2 | 42.6 | 6.9 | 38.6 | 5.1 |
| 14.09.2018 | 49.7 | 5.5 | 42.5 | 5.6 | 45.7 | 7.5 | 48.5 | 7.3 |
| 15.09.2018 | 53.3 | 4.2 | 47.5 | 7.2 | 48.2 | 6.7 | 53.9 | 7.3 |
| 16.09.2018 | 56.0 | 4.0 | 50.0 | 6.9 | 49.7 | 5.9 | 55.1 | 6.6 |
| 17.09.2018 | 57.1 | 4.4 | 50.8 | 6.0 | 50.4 | 5.9 | 53.5 | 5.6 |
| 18.09.2018 | 57.1 | 4.6 | 51.0 | 5.7 | 50.7 | 6.1 | 51.8 | 4.9 |
| 19.09.2018 | 56.5 | 5.2 | 50.9 | 5.4 | 50.8 | 6.4 | 50.2 | 4.5 |
| 20.09.2018 | 55.7 | 5.6 | 50.6 | 5.3 | 50.6 | 6.8 | 48.8 | 4.2 |
| 21.09.2018 | 54.3 | 6.2 | 50.5 | 5.3 | 50.4 | 7.4 | 47.7 | 4.2 |
| 22.09.2018 | 52.7 | 8.3 | 50.4 | 5.3 | 49.9 | 8.7 | 46.6 | 4.4 |
| 23.09.2018 | 51.7 | 8.4 | 49.4 | 5.1 | 48.9 | 10.9 | 44.8 | 4.5 |
| 24.09.2018 | 50.5 | 8.7 | 48.9 | 5.1 | 48.4 | 10.5 | 43.5 | 4.4 |
| 25.09.2018 | 50.0 | 8.8 | 48.5 | 5.2 | 47.3 | 10.9 | 41.8 | 4.2 |
| 26.09.2018 | 38.4 | 14.3 | 36.8 | 13.2 | 38.0 | 13.4 | 31.1 | 10.2 |
| 27.09.2018 | 42.7 | 4.8 | 34.3 | 5.6 | 46.2 | 8.2 | 31.3 | 5.0 |
| 28.09.2018 | 50.0 | 4.1 | 38.3 | 4.8 | 53.8 | 8.4 | 38.9 | 3.7 |
| 29.09.2018 | 53.0 | 3.9 | 38.9 | 4.5 | 55.9 | 6.7 | 42.4 | 3.9 |
| 30.09.2018 | 54.1 | 3.6 | 38.4 | 5.1 | 54.3 | 6.1 | 42.8 | 4.4 |
| 01.10.2018 | 55.0 | 3.5 | 38.5 | 5.6 | 52.5 | 5.9 | 42.8 | 4.8 |
| 02.10.2018 | 54.5 | 3.5 | 37.8 | 6.5 | 50.2 | 6.1 | 42.6 | 5.2 |
| 03.10.2018 | 53.2 | 3.2 | 35.9 | 7.9 | 47.1 | 7.1 | 42.0 | 5.6 |
| 04.10.2018 | 52.8 | 3.5 | 35.9 | 7.2 | 46.4 | 6.4 | 41.9 | 6.0 |
| 05.10.2018 | 51.9 | 4.2 | 36.1 | 6.8 | 46.2 | 5.7 | 42.1 | 6.5 |
| 06.10.2018 | 51.5 | 4.8 | 36.2 | 6.2 | 46.6 | 5.3 | 42.4 | 6.8 |
| 07.10.2018 | 50.6 | 5.1 | 35.7 | 5.7 | 47.2 | 5.3 | 42.4 | 6.8 |
| 08.10.2018 | 48.9 | 5.3 | 34.3 | 5.2 | 47.3 | 5.4 | 41.7 | 6.7 |
| 09.10.2018 | 47.2 | 5.3 | 32.9 | 5.0 | 46.8 | 5.2 | 40.5 | 6.8 |
| 10.10.2018 | 38.9 | 9.7 | 30.4 | 4.5 | 40.1 | 8.7 | 34.4 | 7.8 |
| 11.10.2018 | 30.8 | 4.1 | 27.8 | 2.4 | 34.1 | 4.2 | 30.2 | 1.3 |
| 12.10.2018 | 33.1 | 4.7 | 28.9 | 2.9 | 37.0 | 4.6 | 33.3 | 2.3 |
| 13.10.2018 | 34.5 | 4.9 | 30.0 | 3.1 | 39.0 | 4.1 | 35.9 | 3.8 |
| 14.10.2018 | 35.6 | 5.0 | 31.1 | 3.1 | 39.4 | 4.6 | 37.9 | 5.0 |
| 15.10.2018 | 36.2 | 5.1 | 31.9 | 3.3 | 39.7 | 5.0 | 39.0 | 5.5 |
| 16.10.2018 | 36.6 | 5.2 | 32.6 | 3.5 | 39.7 | 5.4 | 40.0 | 5.8 |
| 17.10.2018 | 36.9 | 5.3 | 33.3 | 3.8 | 39.6 | 5.9 | 40.7 | 5.8 |
| 18.10.2018 | 37.3 | 5.2 | 34.2 | 3.9 | 39.5 | 6.6 | 41.4 | 5.9 |
| 19.10.2018 | 37.2 | 5.4 | 34.7 | 4.2 | 39.6 | 7.4 | 41.7 | 5.9 |
| 20.10.2018 | 36.9 | 5.2 | 34.7 | 4.2 | 39.8 | 7.7 | 41.7 | 5.7 |
| 21.10.2018 | 36.5 | 4.9 | 34.8 | 4.2 | 39.7 | 8.0 | 41.4 | 5.5 |
| 22.10.2018 | 36.3 | 4.4 | 34.7 | 4.0 | 39.5 | 8.1 | 41.4 | 5.2 |
| 23.10.2018 | 36.1 | 4.0 | 34.4 | 4.4 | 39.7 | 8.3 | 37.5 | 7.3 |
| 24.10.2018 | 32.7 | 5.2 | 31.8 | 4.0 | 33.2 | 8.7 | 31.3 | 7.7 |
| 25.10.2018 | 30.0 | 4.6 | 31.3 | 2.2 | 27.5 | 5.6 | 30.4 | 4.3 |
| 26.10.2018 | 29.9 | 4.5 | 31.9 | 2.3 | 27.8 | 6.8 | 33.6 | 5.6 |
| 27.10.2018 | 29.0 | 4.3 | 31.8 | 2.3 | 28.8 | 6.7 | 36.4 | 6.9 |
| 28.10.2018 | 27.8 | 4.4 | 31.3 | 2.5 | 30.0 | 6.7 | 37.1 | 8.3 |
| 29.10.2018 | 26.6 | 4.3 | 30.8 | 2.7 | 31.0 | 6.6 | 37.3 | 8.8 |
| 30.10.2018 | 25.7 | 4.0 | 30.3 | 2.7 | 31.7 | 6.2 | 37.3 | 8.8 |
| 31.10.2018 | 25.2 | 3.5 | 30.4 | 3.0 | 31.5 | 6.4 | 36.7 | 8.7 |
| 01.11.2018 | 24.3 | 3.4 | 29.8 | 2.6 | 30.9 | 6.7 | 33.5 | 9.5 |
| 02.11.2018 | 23.9 | 3.1 | 29.6 | 2.6 | 31.5 | 6.2 | 33.1 | 9.5 |
| 03.11.2018 | 23.1 | 3.2 | 29.1 | 2.5 | 31.2 | 6.4 | 31.6 | 9.8 |
| 04.11.2018 | 22.0 | 3.5 | 28.2 | 2.5 | 31.2 | 6.5 | 30.4 | 9.3 |
| 05.11.2018 | 21.4 | 3.2 | 27.5 | 2.4 | 31.2 | 6.3 | 30.0 | 8.1 |
| 06.11.2018 | 20.9 | 2.9 | 27.2 | 2.4 | 31.5 | 6.0 | 29.2 | 7.9 |
| 07.11.2018 | 20.5 | 2.8 | 26.8 | 2.6 | 31.8 | 5.8 | 28.6 | 7.6 |
| 08.11.2018 | 20.1 | 2.7 | 26.7 | 2.7 | 32.4 | 5.4 | 27.9 | 7.6 |
| 09.11.2018 | 19.3 | 3.2 | 26.1 | 3.1 | 32.5 | 5.6 | 26.6 | 7.9 |
| 10.11.2018 | 18.9 | 2.9 | 25.4 | 2.9 | 32.9 | 5.6 | 26.1 | 7.1 |
| 11.11.2018 | 18.9 | 2.6 | 25.2 | 2.8 | 33.4 | 5.3 | 25.8 | 6.6 |
| 12.11.2018 | 18.7 | 2.5 | 25.0 | 2.9 | 34.0 | 5.2 | 25.1 | 6.5 |
| 13.11.2018 | 18.5 | 2.4 | 25.0 | 3.1 | 34.9 | 4.7 | 24.5 | 6.5 |
| 14.11.2018 | 16.5 | 3.4 | 20.6 | 6.0 | 28.2 | 9.1 | 21.0 | 6.6 |
| 15.11.2018 | 17.3 | 1.1 | 18.0 | 1.6 | 24.3 | 2.2 | 20.2 | 2.0 |
| 16.11.2018 | 18.3 | 0.9 | 19.6 | 1.6 | 26.0 | 2.3 | 21.6 | 2.3 |
| 17.11.2018 | 18.9 | 1.1 | 21.1 | 1.8 | 27.8 | 2.5 | 23.1 | 2.6 |
| 18.11.2018 | 19.5 | 1.4 | 22.7 | 2.2 | 29.7 | 3.1 | 24.6 | 2.9 |
| 19.11.2018 | 20.1 | 1.6 | 24.7 | 3.0 | 31.9 | 4.0 | 26.3 | 2.9 |
| 20.11.2018 | 21.3 | 1.8 | 28.0 | 5.0 | 34.3 | 4.9 | 28.3 | 2.9 |
| 21.11.2018 | 22.4 | 2.2 | 31.7 | 6.9 | 36.4 | 5.4 | 29.9 | 3.2 |
| 22.11.2018 | 23.6 | 2.8 | 35.0 | 8.2 | 38.6 | 5.8 | 30.8 | 3.7 |
| 23.11.2018 | 24.9 | 3.4 | 37.5 | 8.9 | 40.7 | 5.9 | 31.6 | 4.5 |
| 24.11.2018 | 26.4 | 4.3 | 39.1 | 9.1 | 42.3 | 5.8 | 32.1 | 4.7 |
| 25.11.2018 | 28.1 | 5.2 | 39.9 | 8.9 | 43.5 | 5.6 | 32.4 | 4.8 |
| 26.11.2018 | 30.2 | 6.4 | 40.2 | 8.6 | 44.3 | 5.3 | 32.5 | 4.6 |
| 27.11.2018 | 32.5 | 7.4 | 40.2 | 8.4 | 44.7 | 5.2 | 32.4 | 4.5 |
| 28.11.2018 | 34.5 | 8.0 | 39.9 | 8.2 | 45.1 | 5.1 | 32.2 | 4.8 |
| 29.11.2018 | 36.2 | 8.2 | 39.4 | 7.9 | 45.2 | 4.8 | 31.6 | 4.9 |
| 30.11.2018 | 37.1 | 8.2 | 38.7 | 7.4 | 45.0 | 4.6 | 30.5 | 4.6 |
| 01.12.2018 | 37.9 | 7.9 | 37.7 | 7.1 | 45.0 | 4.6 | 29.4 | 4.2 |
| 02.12.2018 | 38.4 | 7.5 | 36.5 | 6.4 | 44.8 | 4.6 | 28.4 | 3.9 |
| 03.12.2018 | 38.7 | 7.5 | 35.5 | 6.0 | 44.4 | 4.6 | 27.6 | 3.7 |
| 04.12.2018 | 38.5 | 7.1 | 34.0 | 5.3 | 43.8 | 4.3 | 26.5 | 3.8 |
| 05.12.2018 | 37.8 | 6.7 | 31.9 | 4.0 | 43.0 | 3.9 | 24.6 | 3.7 |
| 06.12.2018 | 37.2 | 6.2 | 29.9 | 2.9 | 42.0 | 3.7 | 23.1 | 3.4 |
| 07.12.2018 | 36.8 | 6.2 | 28.5 | 2.4 | 41.0 | 3.5 | 22.4 | 2.7 |
| 08.12.2018 | 35.8 | 6.4 | 27.4 | 2.4 | 39.7 | 3.1 | 21.4 | 2.6 |
| 09.12.2018 | 34.2 | 6.0 | 25.4 | 1.7 | 38.0 | 2.4 | 20.0 | 2.7 |
| 10.12.2018 | 32.5 | 5.5 | 23.4 | 1.2 | 36.5 | 2.4 | 18.5 | 3.1 |
| 11.12.2018 | 30.7 | 4.9 | 21.2 | 1.6 | 34.8 | 2.5 | 16.7 | 3.5 |
| 12.12.2018 | 29.1 | 4.4 | 19.6 | 2.0 | 33.1 | 2.3 | 15.5 | 3.4 |
| 13.12.2018 | 27.7 | 4.0 | 18.5 | 2.2 | 31.8 | 2.1 | 14.5 | 3.5 |
| 14.12.2018 | 26.2 | 3.6 | 17.4 | 2.5 | 30.5 | 1.9 | 13.4 | 3.7 |
| 15.12.2018 | 24.6 | 3.3 | 16.3 | 2.8 | 29.1 | 1.8 | 12.2 | 4.1 |
| 16.12.2018 | 23.0 | 3.2 | 15.2 | 3.2 | 27.6 | 1.9 | 10.9 | 4.6 |
| 17.12.2018 | 21.3 | 3.2 | 14.2 | 3.5 | 26.0 | 2.2 | 10.4 | 4.1 |
| 18.12.2018 | 20.0 | 3.0 | 13.6 | 3.5 | 24.5 | 2.3 | 10.3 | 3.4 |
| 19.12.2018 | 18.9 | 2.8 | 13.2 | 3.2 | 23.1 | 2.3 | 9.9 | 3.3 |
| 20.12.2018 | 17.8 | 2.7 | 12.8 | 3.1 | 21.8 | 2.4 | 9.9 | 2.8 |
| 21.12.2018 | 17.0 | 2.5 | 12.6 | 2.8 | 20.7 | 2.3 | 10.1 | 2.3 |
| 22.12.2018 | 16.4 | 2.2 | 12.5 | 2.6 | 19.6 | 2.1 | 9.9 | 2.3 |
| 23.12.2018 | 15.8 | 2.1 | 12.2 | 2.5 | 18.5 | 2.0 | 9.8 | 2.1 |
| 24.12.2018 | 15.1 | 2.1 | 11.9 | 2.5 | 17.5 | 2.0 | 9.1 | 2.9 |
| 25.12.2018 | 14.5 | 2.1 | 11.6 | 2.6 | 16.8 | 2.1 | 9.1 | 2.7 |
| 26.12.2018 | 14.1 | 2.1 | 11.5 | 2.3 | 16.2 | 2.1 | 9.5 | 2.0 |
| 27.12.2018 | 13.9 | 1.8 | 11.7 | 2.0 | 15.6 | 2.0 | 9.9 | 1.6 |
| 28.12.2018 | 13.6 | 1.6 | 11.5 | 1.7 | 15.2 | 1.9 | 10.0 | 1.6 |
| 29.12.2018 | 13.4 | 1.5 | 11.5 | 1.7 | 14.7 | 1.8 | 10.1 | 1.5 |
| 30.12.2018 | 13.1 | 1.4 | 11.5 | 1.7 | 14.3 | 1.7 | 9.9 | 1.7 |
| 31.12.2018 | 12.7 | 1.6 | 11.2 | 1.9 | 13.7 | 1.9 | 9.6 | 2.0 |
| 01.01.2019 | 12.6 | 1.5 | 11.4 | 1.4 | 13.6 | 1.7 | 9.8 | 1.7 |
| 02.01.2019 | 11.9 | 2.0 | 10.6 | 2.1 | 12.9 | 2.1 | 8.4 | 3.3 |
| 03.01.2019 | 11.1 | 2.4 | 9.8 | 2.8 | 12.0 | 2.6 | 7.2 | 4.3 |
| 04.01.2019 | 10.4 | 2.8 | 9.2 | 3.0 | 11.3 | 2.8 | 7.5 | 3.3 |
| 05.01.2019 | 10.3 | 2.4 | 9.3 | 2.6 | 11.0 | 2.6 | 8.3 | 1.9 |
| 06.01.2019 | 10.2 | 2.1 | 9.3 | 2.4 | 10.8 | 2.3 | 7.7 | 2.5 |
| 07.01.2019 | 10.1 | 1.9 | 9.3 | 2.1 | 10.5 | 2.2 | 8.3 | 1.7 |
| 08.01.2019 | 10.3 | 1.6 | 9.6 | 1.7 | 10.5 | 1.8 | 8.3 | 1.6 |
| 09.01.2019 | 9.9 | 1.6 | 9.2 | 2.0 | 10.2 | 1.8 | 7.7 | 2.3 |
| 10.01.2019 | 9.6 | 1.9 | 8.8 | 2.3 | 9.7 | 2.1 | 7.2 | 2.7 |
| 11.01.2019 | 9.4 | 2.0 | 8.6 | 2.3 | 9.3 | 2.1 | 7.5 | 2.1 |
| 12.01.2019 | 9.3 | 1.8 | 8.7 | 2.0 | 9.4 | 1.9 | 7.7 | 1.8 |
| 13.01.2019 | 9.3 | 1.6 | 8.9 | 1.7 | 9.3 | 1.7 | 7.9 | 1.4 |
| 14.01.2019 | 9.2 | 1.5 | 8.7 | 1.7 | 9.2 | 1.6 | 7.3 | 2.3 |
| 15.01.2019 | 8.8 | 1.8 | 8.5 | 1.8 | 8.8 | 1.7 | 7.3 | 1.9 |
| 16.01.2019 | 8.6 | 1.7 | 8.3 | 1.7 | 8.8 | 1.7 | 7.6 | 1.2 |

Table S4. Selective agars for isolation of potential human pathogens. Dilution series of the compost samples were plated on R2A and CHROMagar^TM^ ESBL and transferred to the respective selective agar according to the morphology.

| **Agar** | **Species of interest** | **Incubation** |
| --- | --- | --- |
| ChromoCult® (Merck Millipore) | *Enterococcus* spp. | 37 °C, 24 h, aerobic |
|  | *Enterococcus faecalis* |  |
|  | *Enterococcus faecium* |  |
| Centrimide (Carl Roth GmbH) | *Pseudomonas aeruginosa* | 37 °C, 24 h, aerobic |
| CHROMagar (Mast Diagnostica GmbH) | *Acinetobacter baumannii* | 37 °C, 24 h, aerobic |
| Brain Heart Infusion (BHI; Carl Roth GmbH) | *Staphylococcus aureus* | 37 °C, 24 h, aerobic |
| HiCrome^TM^ *Bacillus* (HiMedia Laboratories) | *Bacillus anthracis* | 37 °C, 24 h, aerobic |
|  | *Bacillus cereus* |  |

Table S5. Antibiotics, their abbreviation and concentration on the disks, as well as breakpoint references for the isolates analyzed. References in brackets for Sulfamethoxazole indicate that breakpoints for Trimethoprim-Sulfamethoxazole were applied instead. Orders in brackets indicate that their breakpoints were used.

| **Antibiotic** | **Abbreviation** | **Concentration [µg]** | **References** | | | | | | |
| --- | --- | --- | --- | --- | --- | --- | --- | --- | --- |
|  |  |  | ***Achromobacter* spp.** | ***Bacillus* spp.** | ***Brucella* spp.** | ***Pseudomonas* spp.** | ***Serratia* spp.** | ***Staphylococcus* spp.** | ***Stenotrophomonas maltophilia*** |
| Ampicillin | AMP | 2 & 10 |  | Mohammadou et al., 2014 |  |  | EUCAST, 2019 (*Enterobacterales*) | EUCAST, 2019 |  |
| Meropenem | MEM | 10 | Almuzara et al., 2010 |  |  | EUCAST, 2019 | EUCAST, 2019 (*Enterobacterales*) |  | EUCAST, 2019 (*Acinetobacter* spp.) |
| Imipenem | IPM | 10 |  | Banerjee et al., 2011 |  |  |  |  |  |
| Ciprofloxacin | CIP | 5 |  | Banerjee et al., 2011 | Gu et al., 2020 | EUCAST, 2019 | EUCAST, 2019 (*Enterobacterales*) | EUCAST, 2019 | EUCAST, 2019 (*Acinetobacter* spp.) |
| Erythromycin | E | 15 |  | Mohammadou et al., 2014 |  |  |  | EUCAST, 2019 |  |
| Vancomycin | VA | 30 |  | Mohammadou et al., 2014 |  |  |  |  |  |
| Clindamycin | DA | 2 |  |  |  |  |  | EUCAST, 2019 |  |
| Doxycycline | DO | 30 | Almuzara et al., 2010 |  | Gu et al., 2020 |  |  |  |  |
| Tetracycline | TE | 30 | Almuzara et al., 2010 | Mohammadou et al., 2014 |  |  |  | EUCAST, 2019 |  |
| Tigecycline | TCG | 15 |  |  |  |  | EUCAST, 2019 (*Enterobacterales*) | EUCAST, 2019 |  |
| Kanamycin | K | 5 & 30 |  | Mohammadou et al., 2014 | Gu et al., 2020 |  |  |  |  |
| Gentamicin | CN | 10 | Almuzara et al., 2010 | Banerjee et al., 2011 | Gu et al., 2020 | EUCAST, 2019 | EUCAST, 2019 (*Enterobacterales*) | EUCAST, 2019 | EUCAST, 2019 (*Acinetobacter* spp.) |
| Sulfamethoxazole | RL | 25 | Almuzara et al., 2010 | Mohammadou et al., 2014 |  |  | EUCAST, 2019 (*Enterobacterales*) | (EUCAST, 2019) | (EUCAST, 2019) |
| Norfloxacin | NOR | 10 |  |  |  |  | EUCAST, 2019 (*Enterobacterales*) | EUCAST, 2019 |  |
| Ofloxacin | OFX | 5 |  | Banerjee et al., 2011 |  |  | EUCAST, 2019 (*Enterobacterales*) | EUCAST, 2019 |  |
| Nitrofurantoin | F | 100 |  |  |  |  | EUCAST, 2019 (*Enterobacterales*) | EUCAST, 2019 |  |

Table S6. Primers for detection of ARGs with target gene, primer sequence, positive control, amplicon size and reference.

| **Resistance gene** | **Primer** | **Sequence (5´→ 3´)** | **Positive control** | **Amplicon size [bp]** | **Reference** |
| --- | --- | --- | --- | --- | --- |
| **Ampicillin** | ampC fw | GTGACCAGATACTGGCCACA | *Enterobacter cloacae* DSM46348 | 822 | Böckelmann et al., 2009 |
|  | ampC rev | TTACTGTAGCGCCTCGAGGA |  |  |  |
| **ß-Lactamase class A** | blaZ fw | TTAAAGTCTTACCGAAAGCAG | *Staphylococcus haemolyticus* VPS617 | 377 | Olsen et al., 2006 |
|  | blaZ rev | TAAGAGATTTGCCTATGCTT |  |  |  |
| **ß-Lactamases (Carbapenemases)** | KPC-Fm | CGTCTAGTTCTGCTGTCTTG | *Escherichia coli* R82 KPC | 798 | Poirel et al., 2011 |
|  | KPC-Rm | CTTGTCATCCTTGTTAGGCG |  |  |  |
|  | IMP-F | GGAATAGAGTGGCTTAAYTCTC | *Klebsiella pneumoniae* R77 IMP-4 | 232 | Ellington et al., 2007 |
|  | IMP-R | GGTTTAAYAAAACAACCACC |  |  | Poirel et al., 2011 |
|  | NDM-F | GGTTTGGCGATCTGGTTTTC | *Escherichia coli* R45 NDM | 621 | Poirel et al., 2011 |
|  | NDM-R | CGGAATGGCTCATCACGATC |  |  |  |
|  | OXA-48-F | GCGTGGTTAAGGATGAACAC | *Klebsiella pneumoniae* R19 OXA-48 | 438 | Poirel et al., 2011 |
|  | OXA-48-R | CATCAAGTTCAACCCAACCG |  |  |  |
| **ß-Lactamases (ESBL)** | CTX-M-F1 | ATAAAACCGGCAGCGGTG | *Escherichia coli* 524_17 | 483 | Leflon-Guibout et al., 2004 |
|  | CTX-M-F2 | GAATTTTGACGATCGGGG |  |  |  |
| **Methicillin** | mecA fw | TAATAGTTGTAGTTGTCGGGTTTG | *Staphylococcus aureus*  DSM13661 | 733 | Böckelmann et al., 2009 |
|  | mecA rev | TAACCTAATAGATGTGAAGTCGCT |  |  |  |
| **Chloramphenicol** | cat_pIP501_ fw | TGGGATAGAAAAGAATATTTTGAACAC | *Enterococcus faecalis* RE25 | 408 | Schiwon et al., 2013 |
|  | cat_pIP501_ rev | TCCAAGGAATCATTGAAATCG |  |  |  |
|  | catLM fw | CTAAAATCAATCCAAGGAATCATCG | *Lactobacillus lactis* K214 | 313 | Schiwon et al., 2013 |
|  | catLM rev | GGATATGAACTGTATCCTGCTTTG |  |  |  |
| **Erythromycin** | ermA fw | ACGATATTCACGGTTTACCCACTTA | *Staphylococcus aureus* MRSA 04-02981 | 610 | Khan et al., 1999 |
|  | ermA rev | AACCAGAAAAACCCTAAAGACACG |  |  |  |
|  | ermB fw | GCATTTAACGACGAAACTGGCT | *Enterococcus gallinarum* | 573 | Böckelmann et al., 2009 |
|  | ermB rev | GACAATACTTGCTCATAAGTAATGGT |  |  |  |
|  | ermC fw | CGTAACTGCCATTGAAATAGACC | *Staphylococcus haemolyticus* VPS617 | 498 | Schiwon et al., 2013 |
|  | ermC rev | TCCTGCATGTTTTAAGGAATTG |  |  |  |
|  | ermD fw | CGGGCAAATATTAGCATAGACG | *Bacillus subtilis* BD1156 | 463 | Schiwon et al., 2013 |
|  | ermD rev | ATTCTGACCATTGCCGAGTC |  |  |  |
|  | ermG fw | TGCAGGGAAAGGTCATTTTAC | *Bacillus subtilis* BD1156 | 483 | Schiwon et al., 2013 |
|  | ermG rev | AACCCATTTCATTACAAAAGTTTC |  |  |  |
| **Fluoroquinolones** | qnrA-F | CAGCAAGAGGATTTCTCACG | *Enterobacter cloacae* No. 562 | 630 | Ciesielczuk et al., 2013 |
|  | qnrA-R | AATCCGGCAGCACTATTACTC |  |  |  |
|  | qnrB-F | GGCTGTCAGTTCTATGATCG | *Klebsiella pneumoniae qnrB1* K8-5 | 488 | Ciesielczuk et al., 2013 |
|  | qnrB-R | GAGCAACGATGCCTGGTAG |  |  |  |
| **Gentamicin** | aac6-aph2a fw | GCCAGAACATGAATTACACGAG | *Staphylococcus aureus* SK5428 | 610 | Schiwon et al., 2013 |
|  | aac6-aph2a rev | CTGTTGTTGCATTTAGTCTTTCC |  |  |  |
|  | aph(2)-Ib fw | AGGATGCCCTTGCATATGATGAAGCGACGT | *Enterococcus faecium* SF11770 | 899 | Perreten et al., 2005 |
|  | aph(2)-Ib rev | ATCAGCATAAGGCGCCGGAAGTAGCAGAAA |  |  |  |
|  | aph(2)-Ic fw | AGCATACAATCCGTCGAGTCGCTTGGTGAG | *Enterococcus gallinarum* SF9117 | 317 | Perreten et al., 2005 |
|  | aph(2)-Ic rev | CTGGCGCTGCAACTTGCTGAGTTCATGAAT |  |  |  |
| **Kanamycin** | aadD_pSK41 fw | TGTCGTTCTGTCCACTCCTG | *Staphylococcus aureus* SK5428 | 525 | Schiwon et al., 2013 |
|  | aadD_pSK41 rev | ATGAATGGACAACCGGTGAG |  |  |  |
|  | aph3-III fw | CCGCTGCGTAAAAGATAC | *Enterococcus faecalis* RE25 | 609 | Schiwon et al., 2013 |
|  | aph3-III rev | GTCATACCACTTGTCCGC |  |  |  |
| **Sulfamethoxazole** | sul1 fw | CACCGGAAACATCGCTGCA | *Escherichia coli* K12 J53 | 158 | Luo et al., 2010 |
|  | sul1 rev | AAGTTCCGCCGCAAGGCT |  |  |  |
|  | sul2 fw | CTCCGATGGAGGCCGGTAT | *Escherichia coli* PS84 | 190 | Luo et al., 2010 |
|  | sul2 rev | GGGAATGCCATCTGCCTTGA |  |  |  |
| **Tetracycline** | tetA-1 | GCTACATCCTGCTTGCCTTC | *Escherichia coli* 524_17 | 210 | Ng et al., 2001 |
|  | tetA-2 | CATAGATCGCCGTGAAGAGG |  |  |  |
|  | tetB-1 | TTGGTTAGGGGCAAGTTTTG | *Escherichia coli* 753_16 | 659 | Ng et al., 2001 |
|  | tetB-2 | GTAATGGGCCAATAACACCG |  |  |  |
|  | tetK_pT181 fw | TTTGAGCTGTCTTGGTTCATTG | *Staphylococcus haemolyticus* VPS G17 | 539 | Schiwon et al., 2013 |
|  | tetK_pT181 rev | AGCCCACCAGAAAACAAACC |  |  |  |
|  | tetL fw | CATTTGGTCTTATTGGATCG | \| *Enterococcus faecium* \| \| --- \| \| SF11770 \| | 475 | Aarestrup et al., 2000 |
|  | tetL rev | ATTACACTTCCGATTTCGG |  |  |  |
|  | tetM fw | GAACTCGAACAAGAGGAAAGC | *Enterococcus faecalis* DS16 | 739 | Olsvik et al., 1995 |
|  | tetM rev | ATGGAAGCCCAGAAAGGAT |  |  |  |
|  | tetS fw | TGGTCAACGGCTTGTCTATG | *Lactobacillus lactis* K214 | 546 | Schiwon et al., 2013 |
|  | tetS rev | AGCCCAGAAAGGATTTGGAG |  |  |  |
| **Vancomycin** | vanB fw | CCCGAATTTCAAATGATTGAAAA | *Enterococcus faecalis* V583 | 456 | Miele et al., 1995 |
|  | vanB rev | CGCCATCCTCCTGCAAAA |  |  |  |
|  | vanC1/C2 fw | ATGGATTGGTA(C/T)T(G/T)GTAT | *Enterococcus casseliflavus* UC73 | 815/82**7** | Depardieu et al., 2004 |
|  | vanC1/C2 rev | TAGCGGGAGTG(A/C)C(C/T)(A/C)GTAA |  |  |  |

Table S7. PCR programs for detection of ARGs. All programs start with an initial denaturation at 95 °C for 2 min and end with a final elongation step at 72 °C for 5 min.

| **Target genes/Primers** | **Temperatures** | **Duration** | **Cycles** |
| --- | --- | --- | --- |
| **ß-Lactamase class A**: *blaZ* | 95 °C | 30 s | 30 |
|  | 60 °C | 45 s |  |
|  | 72 °C | 30 s |  |
| **Carbapenemases**: *blaIMP* | 95 °C | 30 s | 30 |
|  | 50 °C | 30 s |  |
|  | 72 °C | 30 s |  |
| **Methicillin resistance**: *mecA*  **Tetracycline resistance**: *tetA, tetB* | 95 °C | 30 s | 30 |
|  | 60 °C | 30 s |  |
|  | 72 °C | 30 s |  |
| **Erythromycin resistance**: *ermA; ermB; ermC; ermD; ermG*  **Gentamicin resistance**: *aac6-aph2a; aph(2)-Ib: aph(2)-Ic*  **Kanamycin resistance**: *aph3-III* | 95 °C | 30 s | 30 |
|  | 56 °C | 30 s |  |
|  | 72 °C | 30 s |  |
| **Kanamycin resistance**: *aadD_*pSK41 | 95 °C | 30 s | 30 |
|  | 62 °C | 30 s |  |
|  | 72 °C | 45 s |  |
| **Sulfamethoxazole resistance**: *sul1*; *sul2* | 95 °C | 30 s | 30 |
|  | 60 °C | 30 s |  |
|  | 72 °C | 45 s |  |
| **Tetracycline resistance**: *tetK*; *tetL;* *tetM;* *tetS* | 95 °C | 30 s | 30 |
|  | 55 °C | 30 s |  |
|  | 72 °C | 30 s |  |
| **Carbapenemases:** *blaOXA-48* | 95 °C | 30 s | 30 |
|  | 62 °C | 30 s |  |
|  | 72 °C | 30 s |  |
| **Carbapenemases:** *blaKPC, blaNDM-1*  **Vancomycin resistance**: *vanB* | 95 °C | 30 s | 30 |
|  | 57 °C | 30 s |  |
|  | 72 °C | 30 s |  |
| **Ampicillin resistance:** *ampC*  **Chloramphenicol resistance:** *cat_*_pIP501_  **ESBL***: blaCTX-M-15*  **Fluoroquinolone resistance:** *qnrA, qnrB*  **Vancomycin resistance**: *vanC1/C2* | 95 °C | 30 s | 30 |
|  | 54 °C | 30 s |  |
|  | 72 °C | 30 s |  |
| **Chloramphenicol resistance:** *catLM* | 95 °C | 30 s | 30 |
|  | 52 °C | 30 s |  |
|  | 72 °C | 30 s |  |

Table S8. Accession numbers for the 16S rRNA amplicon sequences (library stragegy: WGS, library source: metagenomic, library selection: PCR, library layout: paired, platform: Illumina, instrument model: Illumina MiSeq).

| accession number | study number | bioproject accession number | biosample accession number | library ID | title | design description | file-type | filename | filename2 |
| --- | --- | --- | --- | --- | --- | --- | --- | --- | --- |
| SRR17041405 | SRP347939 | PRJNA782422 | SAMN23382795 | E1-14d_a | 16S rRNA gene-based analysis of the bacterial community of sample E1-14d | Metagenomic DNA was extracted from compost of dry toiletts. The V3-V4 region of 16S rRNA genes was amplified. All samples were amplified in triplicate | fastq | E1-14d_L001_R1_001.fastq.gz | E1-14d_L001_R2_001.fastq.gz |
| SRR17041404 | SRP347939 | PRJNA782422 | SAMN23382798 | E1A_a | 16S rRNA gene-based analysis of the bacterial community of sample E1A | Metagenomic DNA was extracted from compost of dry toiletts. The V3-V4 region of 16S rRNA genes was amplified. All samples were amplified in triplicate | fastq | E1A_L001_R1_001.fastq.gz | E1A_L001_R2_001.fastq.gz |
| SRR17041393 | SRP347939 | PRJNA782422 | SAMN23382806 | E1B-14d_a | 16S rRNA gene-based analysis of the bacterial community of sample E1B-14d | Metagenomic DNA was extracted from compost of dry toiletts. The V3-V4 region of 16S rRNA genes was amplified. All samples were amplified in triplicate | fastq | E1B-14d_L001_R1_001.fastq.gz | E1B-14d_L001_R2_001.fastq.gz |
| SRR17041386 | SRP347939 | PRJNA782422 | SAMN23382793 | E1BA_a | 16S rRNA gene-based analysis of the bacterial community of sample E1BA | Metagenomic DNA was extracted from compost of dry toiletts. The V3-V4 region of 16S rRNA genes was amplified. All samples were amplified in triplicate | fastq | E1BA_L001_R1_001.fastq.gz | E1BA_L001_R2_001.fastq.gz |
| SRR17041385 | SRP347939 | PRJNA782422 | SAMN23382792 | E1Bh_a | 16S rRNA gene-based analysis of the bacterial community of sample E1Bh | Metagenomic DNA was extracted from compost of dry toiletts. The V3-V4 region of 16S rRNA genes was amplified. All samples were amplified in triplicate | fastq | E1Bh_L001_R1_001.fastq.gz | E1Bh_L001_R2_001.fastq.gz |
| SRR17041384 | SRP347939 | PRJNA782422 | SAMN23382794 | E1Bm_a | 16S rRNA gene-based analysis of the bacterial community of sample E1Bm | Metagenomic DNA was extracted from compost of dry toiletts. The V3-V4 region of 16S rRNA genes was amplified. All samples were amplified in triplicate | fastq | E1Bm_L001_R1_001.fastq.gz | E1Bm_L001_R2_001.fastq.gz |
| SRR17041383 | SRP347939 | PRJNA782422 | SAMN23382807 | E1Bv_a | 16S rRNA gene-based analysis of the bacterial community of sample E1Bv | Metagenomic DNA was extracted from compost of dry toiletts. The V3-V4 region of 16S rRNA genes was amplified. All samples were amplified in triplicate | fastq | E1Bv_L001_R1_001.fastq.gz | E1Bv_L001_R2_001.fastq.gz |
| SRR17041382 | SRP347939 | PRJNA782422 | SAMN23382797 | E1h_a | 16S rRNA gene-based analysis of the bacterial community of sample E1h | Metagenomic DNA was extracted from compost of dry toiletts. The V3-V4 region of 16S rRNA genes was amplified. All samples were amplified in triplicate | fastq | E1h_L001_R1_001.fastq.gz | E1h_L001_R2_001.fastq.gz |
| SRR17041381 | SRP347939 | PRJNA782422 | SAMN23382799 | E1m_a | 16S rRNA gene-based analysis of the bacterial community of sample E1m | Metagenomic DNA was extracted from compost of dry toiletts. The V3-V4 region of 16S rRNA genes was amplified. All samples were amplified in triplicate | fastq | E1m_L001_R1_001.fastq.gz | E1m_L001_R2_001.fastq.gz |
| SRR17041380 | SRP347939 | PRJNA782422 | SAMN23382796 | E1v_a | 16S rRNA gene-based analysis of the bacterial community of sample E1v | Metagenomic DNA was extracted from compost of dry toiletts. The V3-V4 region of 16S rRNA genes was amplified. All samples were amplified in triplicate | fastq | E1v_L001_R1_001.fastq.gz | E1v_L001_R2_001.fastq.gz |
| SRR17041403 | SRP347939 | PRJNA782422 | SAMN23382804 | E2A_a | 16S rRNA gene-based analysis of the bacterial community of sample E2A | Metagenomic DNA was extracted from compost of dry toiletts. The V3-V4 region of 16S rRNA genes was amplified. All samples were amplified in triplicate | fastq | E2A_L001_R1_001.fastq.gz | E2A_L001_R2_001.fastq.gz |
| SRR17041402 | SRP347939 | PRJNA782422 | SAMN23382800 | E2BA_a | 16S rRNA gene-based analysis of the bacterial community of sample E2BA | Metagenomic DNA was extracted from compost of dry toiletts. The V3-V4 region of 16S rRNA genes was amplified. All samples were amplified in triplicate | fastq | E2BA_L001_R1_001.fastq.gz | E2BA_L001_R2_001.fastq.gz |
| SRR17041401 | SRP347939 | PRJNA782422 | SAMN23382808 | E2Bh_a | 16S rRNA gene-based analysis of the bacterial community of sample E2Bh | Metagenomic DNA was extracted from compost of dry toiletts. The V3-V4 region of 16S rRNA genes was amplified. All samples were amplified in triplicate | fastq | E2Bh_L001_R1_001.fastq.gz | E2Bh_L001_R2_001.fastq.gz |
| SRR17041400 | SRP347939 | PRJNA782422 | SAMN23382801 | E2Bm_a | 16S rRNA gene-based analysis of the bacterial community of sample E2Bm | Metagenomic DNA was extracted from compost of dry toiletts. The V3-V4 region of 16S rRNA genes was amplified. All samples were amplified in triplicate | fastq | E2Bm_L001_R1_001.fastq.gz | E2Bm_L001_R2_001.fastq.gz |
| SRR17041399 | SRP347939 | PRJNA782422 | SAMN23382809 | E2Bv_a | 16S rRNA gene-based analysis of the bacterial community of sample E2Bv | Metagenomic DNA was extracted from compost of dry toiletts. The V3-V4 region of 16S rRNA genes was amplified. All samples were amplified in triplicate | fastq | E2Bv_L001_R1_001.fastq.gz | E2Bv_L001_R2_001.fastq.gz |
| SRR17041398 | SRP347939 | PRJNA782422 | SAMN23382803 | E2h_a | 16S rRNA gene-based analysis of the bacterial community of sample E2h | Metagenomic DNA was extracted from compost of dry toiletts. The V3-V4 region of 16S rRNA genes was amplified. All samples were amplified in triplicate | fastq | E2h_L001_R1_001.fastq.gz | E2h_L001_R2_001.fastq.gz |
| SRR17041397 | SRP347939 | PRJNA782422 | SAMN23382805 | E2m_a | 16S rRNA gene-based analysis of the bacterial community of sample E2m | Metagenomic DNA was extracted from compost of dry toiletts. The V3-V4 region of 16S rRNA genes was amplified. All samples were amplified in triplicate | fastq | E2m_L001_R1_001.fastq.gz | E2m_L001_R2_001.fastq.gz |
| SRR17041396 | SRP347939 | PRJNA782422 | SAMN23382802 | E2v_a | 16S rRNA gene-based analysis of the bacterial community of sample E2v | Metagenomic DNA was extracted from compost of dry toiletts. The V3-V4 region of 16S rRNA genes was amplified. All samples were amplified in triplicate | fastq | E2v_L001_R1_001.fastq.gz | E2v_L001_R2_001.fastq.gz |

Table S9. MPN data for *E. coli* and *Salmonella* spp. Triplicates of mature compost were used from each pile (front, middle and back).

| ***E. coli* [CFU/g]** | | | | |
| --- | --- | --- | --- | --- |
| replicate | **E1** | **E1-B** | **E2** | **E2-B** |
| front | 92 | <30 | 36 | 160 |
| middle | 36 | 36 | 36 | 36 |
| back | <30 | <30 | <30 | 140 |
| **mean** | **<53** | **<32** | **<34** | **112** |
| ***Salmonella* [CFU/g]** | | | | |
| replicate | **E1** | **E1-B** | **E2** | **E2-B** |
| front | <30 | <30 | <30 | <30 |
| middle | <30 | <30 | <30 | <30 |
| back | 300 | <30 | <30 | <30 |
| **mean** | **<120** | **<30** | **<30** | **<30** |

Table S10. Isolates from mature compost as identified by MALDI-TOF MS biotyping and Sanger sequencing. Piles E1 and E2, replicates without biochar, E1-B, E2-B with biochar. Numbers of the respective species are given as well as biosafety level.

| **Phylum** | **Genus** | **Species** | **Biosafety level** | **E1** | **E2** | **E1-B** | **E2-B** | **Number of Isolates** |
| --- | --- | --- | --- | --- | --- | --- | --- | --- |
| *Actinobacteria* | *Cellulosimicrobium* | *cellulans* | 1 | 0 | 1 | 0 | 0 | 1 |
|  | *Dermacoccus* | *nishinomiyaensis* | 1 | 1 | 0 | 0 | 0 | 1 |
|  | *Rhodococcus* | *rhodochrous* | 1 | 1 | 0 | 0 | 0 | 1 |
|  | *Streptomyces* | *thermocoprophilus* | 1 | 1 | 0 | 0 | 0 | 1 |
|  | *Streptomyces* | *thermocarboxydus* | 1 | 1 | 0 | 0 | 0 | 1 |
| *Firmicutes* | *Bacillus* | *aerius* | 1 | 1 | 0 | 0 | 0 | 1 |
|  | *Bacillus* | *albus* | 1 | 0 | 0 | 5 | 1 | 6 |
|  | *Bacillus* | *altitudinis* | 1 | 0 | 0 | 1 | 0 | 1 |
|  | *Bacillus* | *cereus* | 2 | 0 | 0 | 1 | 0 | 1 |
|  | *Bacillus* | *licheniformis* | 1 | 1 | 3 | 1 | 1 | 6 |
|  | *Bacillus* | *megaterium* | 1 | 1 | 0 | 0 | 1 | 2 |
|  | *Bacillus* | *pumilus* | 1 | 0 | 0 | 3 | 0 | 3 |
|  | *Bacillus* | *safensis* | 1 | 0 | 0 | 1 | 0 | 1 |
|  | *Bacillus* | *subtilis* | 1 | 22 | 9 | 18 | 4 | 53 |
|  | *Lysinibacillus* | *fusiformis* | 1 | 0 | 0 | 1 | 0 | 1 |
|  | *Staphylococcus* | *epidermidis* | 2 | 0 | 1 | 0 | 0 | 1 |
|  | *Staphylococcus* | *haemolyticus* | 2 | 0 | 0 | 0 | 1 | 1 |
| *Proteobacteria* | *Achromobacter* | *denitrificans* | 2 | 0 | 2 | 0 | 0 | 2 |
|  | *Achromobacter* | *mucicolens* | 2 | 0 | 0 | 0 | 2 | 2 |
|  | *Achromobacter* | *spanius* | 1 | 0 | 0 | 0 | 1 | 1 |
|  | *Bordetella* | *petrii* | 1 | 4 | 5 | 4 | 1 | 14 |
|  | *Brucella* | *daejeonensis* | 1 | 0 | 0 | 2 | 0 | 2 |
|  | *Brucella* | *intermedia* | 2 | 12 | 13 | 9 | 8 | 42 |
|  | *Brucella* | *tritici* | 1 | 1 | 1 | 3 | 0 | 5 |
|  | *Castellaniella* | *daejeonensis* | 1 | 1 | 0 | 0 | 0 | 1 |
|  | *Pseudomonas* | *aeruginosa* | 2 | 1 | 1 | 0 | 2 | 4 |
|  | *Pseudomonas* | *khazarica* | 1 | 0 | 0 | 0 | 1 | 1 |
|  | *Pseudomonas* | *koreensis* | 1 | 1 | 0 | 0 | 0 | 1 |
|  | *Pseudomonas* | *mendocina* | 2 | 3 | 1 | 0 | 2 | 6 |
|  | *Pseudomonas* | *monteilii* | 1 | 0 | 0 | 0 | 1 | 1 |
|  | *Pseudomonas* | *pseudoalcaligenes* | 1 | 0 | 1 | 0 | 0 | 1 |
|  | *Pseudomonas* | *“sediminis”* | * | 0 | 2 | 0 | 0 | 2 |
|  | *Pseudomonas* | *“sihuiensis”* | * | 1 | 1 | 0 | 2 | 4 |
|  | *Serratia* | *fonticola* | 1 | 3 | 0 | 1 | 1 | 5 |
|  | *Serratia* | *marcescens* | 2 | 3 | 0 | 6 | 2 | 11 |
|  | *Serratia* | *nematodiphila* | 1 | 0 | 0 | 1 | 0 | 1 |
|  | *Serratia* | *“surfactantfaciens”* | * | 0 | 0 | 4 | 2 | 6 |
|  | *Serratia* | *ureilytica* | 1 | 0 | 0 | 1 | 0 | 1 |
|  | *Stenotrophomonas* | *maltophilia* | 2 | 0 | 2 | 1 | 0 | 3 |
|  | *Stenotrophomonas* | *pavanii* | 1 | 0 | 2 | 0 | 0 | 2 |
| Total number | | | | 59 | 45 | 63 | 33 | 200 |

*safety level could not be assigned, since the species has not been validly published (*Taxonomy Browser (Pseudomonas sediminis)*; *Taxonomy Browser (Pseudomonas sihuiensis)*; *Taxonomy Browser (Serratia surfactantfaciens)*)

Table S11. Antibiotic resistance profile of BSL-2 isolates. Antibiotics in brackets indicate intermediate resistance (susceptibility) at increased exposure according to EUCAST (2019).

| Origin | Species | Resistances* | number of isolates | Label |
| --- | --- | --- | --- | --- |
| E1 (front) | *Brucella intermedia* | (CIP5), K30 | 1 | E1_f_81 |
|  | *Brucella intermedia* | (CIP5), (CN10), K30 | 1 | E1_f_458 |
|  | *Pseudomonas aeruginosa* | - | 1 | E1_f_324 |
|  | *“Pseudomonas sihuiensis”* | - | 1 | E1_f_7 |
|  | *Serratia marcescens* | AMP10, F100, (MEM10), RL25, TCG15 | 1 | E1_f_336 |
|  | *Serratia marcescens* | AMP10, RL25 | 1 | E1_f_337 |
|  | *Serratia marcescens* | AMP10, CIP5, (CN10), F100, (MEM10), (OFX5), RL25, TCG15 | 1 | E1_f_338 |
| E1 (back) | *Brucella intermedia* | (CIP5), (CN10), K30 | 5 | E1_b_21, E1_b_23, E1_b_25, E1_b_30, E1_b_360 |
|  | *Brucella intermedia* | (CIP5), K30 | 5 | E1_b_22, E1_b_26, E1_b_27, E1_b_28, E1_b_29 |
|  | *Pseudomonas mendocina* | - | 2 | E1_b_102, E1_b_103 |
|  | *Pseudomonas mendocina* | (MEM10) | 1 | E1_b_191 |
| E2 (front) | *Brucella intermedia* | (CIP5), (CN10), K30 | 7 | E2_f_11, E2_f_12, E2_f_13, E2_f_14, E2_f_15, E2_f_87, E2_f_88 |
|  | *Brucella intermedia* | (CIP5), K30 | 1 | E2_f_86 |
|  | *Brucella intermedia* | CIP5, (DO30), K30 | 1 | E2_f_96 |
|  | *Pseudomonas mendocina* | - | 1 | E2_f_20 |
|  | *Staphylococcus epidermidis* | RL25 | 1 | E2_f_89 |
| E2 (back) | *Achromobacter denitrificans* | (CN10), (MEM10), RL25 | 1 | E2_b_114 |
|  | *Achromobacter denitrificans* | (CN10), RL25 | 1 | E2_b_115 |
|  | *Brucella intermedia* | (CIP5), (CN10), K30 | 2 | E2_b_33, E2_b_123 |
|  | *Brucella intermedia^1^* | (CN10), K30 | 1 | E2_b_111 |
|  | *Brucella intermedia* | CIP5, DO30, K30 | 1 | E2_b_122 |
|  | *Pseudomonas aeruginosa* | - | 1 | E2_b_322 |
|  | *“Pseudomonas sediminis”* | - | 2 | E2_b_112, E2_b_113 |
|  | *“Pseudomonas sihuiensis”^3^* | - | 1 | E2_b_31 |
|  | *Stenotrophomonas maltophilia* | AMP2, CIP5, CN10, DA2, F100, K5, KF30^#^, MEM10, OX5^#^, RL25, VA30 | 1 | E2_b_35 |
|  | *Stenotrophomonas maltophilia* | AMP2, CIP5, CN10, DA2, F100, K5, KF30^#^, MEM10, OX1^#^, VA30 | 1 | E2_b_38 |
| E1-B (front) | *Bacillus cereus* | AMP10, (RL25), VA30^+^ | 1 | E1B_f_125 |
|  | *Brucella intermedia* | (CIP5), K30 | 3 | E1B_f_45, E1B_f_47, E1B_f_48 |
|  | *Brucella intermedia* | (CIP5), (CN10), K30 | 3 | E1B_f_46, E1B_f_370, E1B_f_468 |
|  | *Brucella intermedia* | CIP5, (DO30), K30 | 1 | E1B_f_130 |
|  | *Serratia marcescens* | AMP10, (MEM10), RL25 | 1 | E1B_f_369 |
|  | *Serratia marcescens* | AMP10, RL25 | 1 | E1B_f_373 |
|  | *Serratia marcescens^2^* | AMP10, RL25, TCG15 | 1 | E1B_f_376 |
|  | *Serratia marcescens* | AMP10, (MEM10), RL25, TCG15 | 1 | E1B_f_378 |
|  | *Serratia marcescens* | AMP10, CN10, F100, OFX5, RL25, TCG15 | 1 | E1B_f_381 |
|  | *Serratia marcescens* | AMP10, (MEM10), RL25 | 1 | E1B_f_382 |
|  | *“Serratia surfactantfaciens” ^4^* | AMP10, F100, RL25, TGC15 | 2 | E1B_f_368, E1B_f_380 |
|  | *“Serratia surfactantfaciens”* | AMP10, (MEM10), RL25 | 1 | E1B_f_383 |
| E1-B (back) | *Brucella intermedia* | (CIP5), K30 | 1 | E1B_b_57 |
|  | *Brucella intermedia* | (CIP5), (CN10), K30 | 1 | E1B_b_58 |
|  | *“Serratia surfactantfaciens”* | F100, (OFX5), RL25 | 1 | E1B_b_425 |
|  | *Stenotrophomonas maltophilia* | AMP2, CIP5, CN10, DA2, F100, K5, KF30^#^, MEM10, OX5^#^, RL25, VA30 | 1 | E1B_b_55 |
| E2-B (front) | *Achromobacter mucicolens* | (CN10), (TE30) | 1 | E2B_f_144 |
|  | *Achromobacter mucicolens* | (CN10), (MEM10), RL25 | 1 | E2B_f_63 |
|  | *Brucella intermedia* | (CIP5), (CN10), K30 | 7 | E2B_f_64, E2B_f_67, E2B_f_69, E2B_f_70, E2B_f_140, E2B_f_141, E2B_f_143 |
|  | *Pseudomonas aeruginosa* | - | 1 | E2B_f_320 |
|  | *Pseudomonas mendocina* | MEM10 | 1 | E2B_f_65 |
|  | *Pseudomonas mendocina* | - | 1 | E2B_f_66 |
|  | *Serratia marcescens* | AMP10, F100, RL25 | 1 | E2B_f_402 |
|  | *Serratia marcescens* | AMP10, (CN10), F100, (NOR10), (OFX5), RL25, TCG15 | 1 | E2B_f_404 |
|  | *“Serratia surfactantfaciens”* | F100, RL25 | 1 | E2B_f_395 |
|  | *“Serratia surfactantfaciens”* | AMP10, RL25 | 1 | E2B_f_403 |
|  | *Staphylococcus haemolyticus* | AMP2, CN10, DA2, E15, F100, OFX5, RL25, TCG15, TE30 | 1 | E2B_f_329 |
| E2-B (back) | *Brucella intermedia* | (CIP5), (CN10), K30 | 1 | E2B_b­_148 |
|  | *Pseudomonas aeruginosa* | - | 1 | E2B_b­_321 |
|  | *“Pseudomonas sihuiensis”* | - | 2 | E2B_b­_75, E2B_b­_76 |

*Antibiotic susceptibility testing via disc diffusion assay according to EUCAST (2019); AMP, ampicillin; CIP, ciprofloxacin; CN, gentamicin; DA, clindamycin; DO, doxycycline; E, erythromycin; F, nitrofurantoin; K, kanamycin; KF, cephalothin; MEM, meropenem; NOR, norfloxacin; OFX, ofloxacin; OX, oxacillin; RL, sulfamethoxazole; TCG, tigecycline; TE, tetracycline; VA, vancomycin; numbers indicate the antibiotic disk content in [µg]

^+^zone diameters <17 mm were defined as resistant, but according to the breakpoint tables MIC should be determined in this case

^#^no breakpoints available; isolates did not exhibit an inhibition zone

^1^ciprofloxacin results were inconsistent with three independent tests interpreted as intermediate and resistant respectively

^2^ofloxacin results were inconsistent with three independent tests interpreted as sensitive and intermediate respectively

*^3^Pseudomonas* *sihuiensis* (BLAST % Ident. 100.00%)/*Pseudomonas* *chengduensis* (100.00%)/*Pseudomonas alcaliphila* (100.00%)/*Pseudomonas oleovorans* (100.00%)

*^4^”Serratia surfactantfaciens”* (BLAST % Ident. 100.00%)/*Serratia nematodiphila* (100.00%)

Table S12. BLASTn percent identity values, query coverage, as well as reference accession numbers of isolates of the same origin with the same phenotypic resistance profiles. Different BLAST scores could indicate different strains.

| Origin | Species | Label | Resistances* | number of isolates | BLAST % Ident. | BLAST Query coverage | BLAST Acc. No. |
| --- | --- | --- | --- | --- | --- | --- | --- |
| E1 (back) | *Brucella intermedia* | E1_b_21, E1_b_25 | (CIP5), (CN10), K30 | 2 | 99.86% | 100% | [NR_113812.1](https://www.ncbi.nlm.nih.gov/nucleotide/NR_113812.1?report=genbank&log$=nucltop&blast_rank=1&RID=MZ31987K016) |
|  | *Brucella intermedia* | E1_b_23, E1_b_30 | (CIP5), (CN10), K30 | 2 | 99.44% | 100% | [NR_113812.1](https://www.ncbi.nlm.nih.gov/nucleotide/NR_113812.1?report=genbank&log$=nucltop&blast_rank=1&RID=MZ31987K016) |
|  | *Brucella intermedia* | E1_b_360 | (CIP5), (CN10), K30 | 1 | 100.00% | 100% | [NR_113812.1](https://www.ncbi.nlm.nih.gov/nucleotide/NR_113812.1?report=genbank&log$=nucltop&blast_rank=1&RID=MZ31987K016) |
|  | *Brucella intermedia* | E1_b_22, E1_b_26, E1_b_27, E1_b_28, E1_b_29 | (CIP5), K30 | 5 | 99.59% | 100% | [NR_113812.1](https://www.ncbi.nlm.nih.gov/nucleotide/NR_113812.1?report=genbank&log$=nucltop&blast_rank=1&RID=MZ31987K016) |
|  | *Pseudomonas mendocina* | E1_b_102, E1_b_103 | - | 2 | 98.51% | 99% | [LR134290.1](https://www.ncbi.nlm.nih.gov/nucleotide/LR134290.1?report=genbank&log$=nucltop&blast_rank=2&RID=MZ4766BW013) |
| E2 (front) | *Brucella intermedia* | E2_f_11, E2_f_12, E2_f_13, E2_f_14, E2_f_15, E2_f_87 | (CIP5), (CN10), K30 | 6 | 99.89% | 100% | [NR_113812.1](https://www.ncbi.nlm.nih.gov/nucleotide/NR_113812.1?report=genbank&log$=nucltop&blast_rank=1&RID=MZ1R1ZPB013) |
|  | *Brucella intermedia* | E2_f_88 | (CIP5), (CN10), K30 | 1 | 99.79% | 100% | [NR_113812.1](https://www.ncbi.nlm.nih.gov/nucleotide/NR_113812.1?report=genbank&log$=nucltop&blast_rank=1&RID=MZ1R1ZPB013) |
| E2 (back) | *Brucella intermedia* | E2_b_33, E2_b_123 | (CIP5), (CN10), K30 | 2 | 99.89% | 100% | [NR_113812.1](https://www.ncbi.nlm.nih.gov/nucleotide/NR_113812.1?report=genbank&log$=nucltop&blast_rank=1&RID=MZ15CZ6T013) |
|  | *Brucella intermedia^1^* | E2_b_111 | (CN10), K30 | 1 | 99.89% | 100% | [NR_113812.1](https://www.ncbi.nlm.nih.gov/nucleotide/NR_113812.1?report=genbank&log$=nucltop&blast_rank=1&RID=MZ15KUB9013) |
|  | *“Pseudomonas sediminis”* | E2_b_112, E2_b_113 | - | 2 | 99.75% | 100% | [KP319033.1](https://www.ncbi.nlm.nih.gov/nucleotide/KP319033.1?report=genbank&log$=nucltop&blast_rank=1&RID=MZ0R9VF0013) |
| E1-B (front) | *Brucella intermedia* | E1B_f_45 | (CIP5), K30 | 1 | 98.30% | 100% | [NR_113812.1](https://www.ncbi.nlm.nih.gov/nucleotide/NR_113812.1?report=genbank&log$=nucltop&blast_rank=1&RID=MYZT0XEX013) |
|  | *Brucella intermedia* | E1B_f_47 | (CIP5), K30 | 1 | 98.14% | 100% | [NR_113812.1](https://www.ncbi.nlm.nih.gov/nucleotide/NR_113812.1?report=genbank&log$=nucltop&blast_rank=1&RID=MYZTSJEG013) |
|  | *Brucella intermedia* | E1B_f_48 | (CIP5), K30 | 1 | 100.00% | 100% | [NR_113812.1](https://www.ncbi.nlm.nih.gov/nucleotide/NR_113812.1?report=genbank&log$=nucltop&blast_rank=1&RID=MYZTSJEG013) |
|  | *Brucella intermedia* | E1B_f_46, E1B_f_370 | (CIP5), (CN10), K30 | 2 | 99.59% | 100% | [NR_113812.1](https://www.ncbi.nlm.nih.gov/nucleotide/NR_113812.1?report=genbank&log$=nucltop&blast_rank=1&RID=MYYVVY9Y013) |
|  | *Brucella intermedia* | E1B_f_468 | (CIP5), (CN10), K30 | 1 | 99.90% | 100% | [NR_113812.1](https://www.ncbi.nlm.nih.gov/nucleotide/NR_113812.1?report=genbank&log$=nucltop&blast_rank=1&RID=MYYX1F1Z013) |
|  | *“Serratia surfactantfaciens”*^2^ | E1B_f_368, E1B_f_380 | AMP10, F100, RL25, TGC15 | 2 | 100.00% | 100% | CP016948.1 |
| E2-B (front) | *Brucella intermedia* | E2B_f_64, E2B_f_67, E2B_f_69, E2B_f_70, E2B_f_140, E2B_f_141, E2B_f_143 | (CIP5), (CN10), K30 | 7 | 99.89% | 100% | [NR_113812.1](https://www.ncbi.nlm.nih.gov/nucleotide/NR_113812.1?report=genbank&log$=nucltop&blast_rank=1&RID=MYWMTC4G013) |
| E2-B (back) | *“Pseudomonas sihuiensis”* | E2B_b_75, E2B_b_76 | - | 2 | 99.68% | 100% | [LT629797.1](https://www.ncbi.nlm.nih.gov/nucleotide/LT629797.1?report=genbank&log$=nucltop&blast_rank=1&RID=MYV3R0XJ013) |

*Antibiotic susceptibility testing via disc diffusion assay according to EUCAST (2019); AMP, ampicillin; CIP, ciprofloxacin; CN, gentamicin; DA, clindamycin; DO, doxycycline; E, erythromycin; F, nitrofurantoin; K, kanamycin; KF, cephalothin; MEM, meropenem; NOR, norfloxacin; OFX, ofloxacin; OX, oxacillin; RL, sulfamethoxazole; TCG, tigecycline; TE, tetracycline; VA, vancomycin; numbers indicate the antibiotic disk content in [µg]

^1^ciprofloxacin results were inconsistent with three independent tests interpreted as intermediate and resistant respectively

^2^*”Serratia surfactantfaciens”* (BLAST % Ident. 100.00%)/*Serratia nematodiphila* (100.00%)

References

Aarestrup, F. M., Agerso, Y., Gerner-Smidt, P., Madsen, M., & Jensen, L. B. (2000). Comparison of antimicrobial resistance phenotypes and resistance genes in *Enterococcus faecalis* and *Enterococcus faecium* from humans in the community, broilers, and pigs in Denmark. *Diagnostic Microbiology and Infectious Disease*, *37*(2), 127–137. https://doi.org/10.1016/S0732-8893(00)00130-9

Almuzara, M., Limansky, A., Ballerini, V., Galanternik, L., Famiglietti, A., & Vay, C. (2010). In vitro susceptibility of *Achromobacter* spp. isolates: comparison of disk diffusion, Etest and agar dilution methods. *International Journal of Antimicrobial Agents*, *35*(1), 68–71. https://doi.org/10.1016/j.ijantimicag.2009.08.015

Banerjee, M., Nair, G. B., & Ramamurthy, T. (2011). Phenotypic & genetic characterization of *Bacillus cereus* isolated from the acute diarrhoeal patients. *Indian Journal of Medical Research*, *133*(1), 88–95.

Böckelmann, U., Dörries, H. H., Ayuso-Gabella, M. N., De Marçay, M. S., Tandoi, V., Levantesi, C., Masciopinto, C., Houtte, E. Van, Szewzyk, U., Wintgens, T., & Grohmann, E. (2009). Quantitative PCR monitoring of antibiotic resistance genes and bacterial pathogens in three european artificial groundwater recharge systems. *Applied and Environmental Microbiology*, *75*(1), 154–163. https://doi.org/10.1128/AEM.01649-08

Ciesielczuk, H., Hornsey, M., Choi, V., Woodford, N., & Wareham, D. W. (2013). Development and evaluation of a multiplex PCR for eight plasmid-mediated quinolone-resistance determinants. *Journal of Medical Microbiology*, *62*, 1823–1827. https://doi.org/10.1099/jmm.0.064428-0

Depardieu, F., Perichon, B., & Courvalin, P. (2004). Detection of the *van* alphabet and identification of enterococci and staphylococci at the species level by multiplex PCR. *Journal of Clinical Microbiology*, *42*(12), 5857–5860. https://doi.org/10.1128/JCM.42.12.5857-5860.2004

Ellington, M. J., Kistler, J., Livermore, D. M., & Woodford, N. (2007). Multiplex PCR for rapid detection of genes encoding acquired metallo-β-lactamases. *Journal of Antimicrobial Chemotherapy*, *59*, 321–322. https://doi.org/10.1093/jac/dkl481

EUCAST. (2019). *The European Committee on Antimicrobial Susceptibility Testing. Breakpoint tables for interpretation of MICs and zone diameters. Version 9.0, 2019.* http://www.eucast.org/fileadmin/src/media/PDFs/EUCAST_files/Breakpoint_tables/v_5.0_Breakpoint_Table_01.pdf

Gu, S., Hou, R., Gao, S., Sun, Z., Li, X., Zhai, L., Jin, Y., Zhu, Q., Liao, Y., & Tian, K. (2020). First Isolation and Characterization of *Ochrobactrum anthropi* from Pig. *Engineering*, *6*(1), 49–55. https://doi.org/10.1016/j.eng.2019.08.014

Khan, S. A., Nawaz, M. S., Khan, A. A., & Cerniglia, C. E. (1999). Simultaneous detection of erythromycin-resistant methylase genes *ermA* and *ermC* from *Staphylococcus* spp. by multiplex-PCR. *Molecular and Cellular Probes*, *13*(5), 381–387. https://doi.org/10.1006/mcpr.1999.0265

Leflon-Guibout, V., Jurand, C., Bonacorsi, S., Espinasse, F., Guelfi, M. C., Duportail, F., Heym, B., Bingen, E., & Nicolas-Chanoine, M. H. (2004). Emergence and spread, of three clonally related virulent isolates of *CTX-M-15*-producing *Escherichia coli* with variable resistance to aminoglycosides and tetracycline in a French geriatric hospital. *Antimicrobial Agents and Chemotherapy*, *48*(10), 3736–3742. https://doi.org/10.1128/AAC.48.10.3736-3742.2004

Luo, Y., Mao, D., Rysz, M., Zhou, Q., Zhang, H., Xu, L., & Alvarez, P. J. J. (2010). Trends in antibiotic resistance genes occurrence in the Haihe River, China. *Environmental Science and Technology*, *44*(19), 7220–7225. https://doi.org/10.1021/es100233w

Miele, A., Bandera, M., & Goldstein, B. P. (1995). Use of primers selective for vancomycin resistance genes to determine van genotype in enterococci and to study gene organization in VanA isolates. *Antimicrobial Agents and Chemotherapy*, *39*(8), 1772–1778. https://doi.org/10.1128/AAC.39.8.1772

Mohammadou, B.-A., Le Blay, G., Mbofung, C. M., & Barbier, G. (2014). Antimicrobial activities, toxinogenic potential and sensitivity to antibiotics of *Bacillus* strains isolated from Mbuja, an *Hibiscus sabdariffa* fermented seeds from Cameroon. *African Journal of Biotechnology*, *13*(35), 3617–3627. https://doi.org/10.5897/ajb2014.13907

Ng, L. K., Martin, I., Alfa, M., & Mulvey, M. (2001). Multiplex PCR for the detection of tetracycline resistant genes. *Molecular and Cellular Probes*, *15*(4), 209–215. https://doi.org/10.1006/mcpr.2001.0363

Olsen, J. E., Christensen, H., & Aarestrup, F. M. (2006). Diversity and evolution of *blaZ* from *Staphylococcus aureus* and coagulase-negative staphylococci. *Journal of Antimicrobial Chemotherapy*, *57*(3), 450–460. https://doi.org/10.1093/jac/dki492

Olsvik, B., Olsen, I., & Tenover, F. C. (1995). Detection of *tet(M)* and *tet(Q)* using the polymerase chain reaction in bacteria isolated from patients with periodontal disease. *Oral Microbiology and Immunology*, *10*(2), 87–92. https://doi.org/10.1111/j.1399-302X.1995.tb00124.x

Perreten, V., Vorlet-Fawer, L., Slickers, P., Ehricht, R., Kuhnert, P., & Frey, J. (2005). Microarray-based detection of 90 antibiotic resistance genes of gram-positive bacteria. *Journal of Clinical Microbiology*, *43*(5), 2291–2302. https://doi.org/10.1128/JCM.43.5.2291-2302.2005

Poirel, L., Walsh, T. R., Cuvillier, V., & Nordmann, P. (2011). Multiplex PCR for detection of acquired carbapenemase genes. *Diagnostic Microbiology and Infectious Disease*, *70*(1), 119–123. https://doi.org/10.1016/j.diagmicrobio.2010.12.002

Schiwon, K., Arends, K., Rogowski, K. M., Fürch, S., Prescha, K., Sakinc, T., Van Houdt, R., Werner, G., & Grohmann, E. (2013). Comparison of Antibiotic Resistance, Biofilm Formation and Conjugative Transfer of *Staphylococcus* and *Enterococcus* Isolates from International Space Station and Antarctic Research Station Concordia. *Microbial Ecology*, *65*(3), 638–651. https://doi.org/10.1007/s00248-013-0193-4

*Taxonomy browser (Pseudomonas sediminis)*. Retrieved September 30, 2021, from https://www.ncbi.nlm.nih.gov/Taxonomy/Browser/wwwtax.cgi

*Taxonomy browser (Pseudomonas sihuiensis)*. Retrieved September 30, 2021, from https://www.ncbi.nlm.nih.gov/Taxonomy/Browser/wwwtax.cgi

*Taxonomy browser (Serratia surfactantfaciens)*. Retrieved September 30, 2021, from https://www.ncbi.nlm.nih.gov/Taxonomy/Browser/wwwtax.cgi
